# Defective Homologous Recombination Repair By Up‐Regulating Lnc‐HZ10/Ahr Loop in Human Trophoblast Cells Induced Miscarriage

**DOI:** 10.1002/advs.202207435

**Published:** 2024-01-29

**Authors:** Weina Chen, Chenyang Mi, Ying Zhang, Yang Yang, Wenxin Huang, Zhongyan Xu, Jingsong Zhao, Rong Wang, Manli Wang, Shukun Wan, Xiaoqing Wang, Huidong Zhang

**Affiliations:** ^1^ Research Center for Environment and Female Reproductive Health The Eighth Affiliated Hospital Sun Yat‐sen University Shenzhen 518033 China; ^2^ Key Laboratory of Environment and Female Reproductive Health West China School of Public Health & West China Fourth Hospital Sichuan University Chengdu 610041 China

**Keywords:** environmental BaP or BPDE, female trophoblast cells, homologous recombination, miscarriage, non‐coding RNA lnc‐HZ10

## Abstract

Human trophoblast cells are crucial for healthy pregnancy. However, whether the defective homologous recombination (HR) repair of dsDNA break (DSB) in trophoblast cells may induce miscarriage is completely unknown. Moreover, the abundance of BRCA1 (a crucial protein for HR repair), its recruitment to DSB foci, and its epigenetic regulatory mechanisms, are also fully unexplored. In this work, it is identified that a novel lnc‐HZ10, which is highly experssed in villous tissues of recurrent miscarriage (RM) vs their healthy control group, suppresses HR repair of DSB in trophoblast cell. Lnc‐HZ10 and AhR (aryl hydrocarbon receptor) form a positive feedback loop. AhR acts as a transcription factor to promote lnc‐HZ10 transcription. Meanwhile, lnc‐HZ10 also increases AhR levels by suppressing its CUL4B‐mediated ubiquitination degradation. Subsequently, AhR suppresses BRCA1 transcription; and lnc‐HZ10 (mainly 1‐447 nt) interacts with γ‐H2AX; and thus, impairs its interactions with BRCA1. BPDE exposure may trigger this loop to suppress HR repair in trophoblast cells, possibly inducing miscarriage. Knockdown of murine Ahr efficiently recovers HR repair in placental tissues and alleviates miscarriage in a mouse miscarriage model. Therefore, it is suggested that AhR/lnc‐HZ10/BRCA1 axis may be a promising target for alleviation of unexplained miscarriage.

## Graphical abstract

A novel lnc‐HZ10 suppresses homologous recombination repair of dsDNA break in human trophoblast cells. Lnc‐HZ10 and AhR form a positive feedback loop. AhR suppresses BRCA1 transcription; and lnc‐HZ10 interacts with γ‐H2AX to impair the interactions of γ‐H2AX with BRCA1. AhR/lnc‐HZ10/BRCA1 axis can be considered as a promising target for alleviation of unexplained miscarriage.

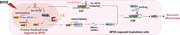

## 1 Introduction

Spontaneous miscarriage occurs in ≈15–25% of pregnant women and recurrent miscarriage (RM, two or more consecutive spontaneous miscarriages) occurs in ≈1–5% of pregnant women.^[^ ^1^, ^2^ ^]^ RM not only reduces population growth rate but also produces severe social, emotional, and psychological hurts on the women and their families.^[^ ^3^ ^]^ In general, chromosome abnormalities; genetic causes; and endocrine, autoimmune, and thrombotic abnormalities are considered as the causes of RM.^[^ ^4^ ^]^ However, there are still 50% RM with unexplained causes, which are called unexplained RM.^[^ ^5^ ^]^ Extravillous trophoblast cells take important roles in embryo implantation and the formation of functional placenta.^[^ ^6^ ^]^ Dysfunctions of human trophoblast cells may lead to various adverse pregnancy outcomes, including miscarriage. In our recent work, we have identified that inhibition in trophablast cell proliferation, migration/invasion or the increase in trophoblast cell apoptosis is associated with RM.^[^ ^7^, ^8^, ^9^, ^10^, ^11^, ^12^, ^13^, ^14^, ^15^ ^]^ However, whether other dysfunctions of human trophoblast cells might also induce miscarriage should be further explored.

DNA double‐strand break (DSB) could be generated by endogenous factors (e.g., oxidative stress,^[^ ^16^ ^]^ hydrolysis reaction,^[^ ^17^ ^]^ inflammation,^[^ ^18^ ^]^ etc.) or exogenous environmental factors (e.g., ultraviolet, ionizing radiation);^[^ ^19^ ^]^ and thus, may lead to genomic instability, chromosomal aberration, and cell death.^[^ ^20^ ^]^ In trophoblasts of mice, the increased levels of DSB are associated with placental proliferation inhibition and fetal intrauterine growth restriction.^[^ ^21^ ^]^ Compared to non‐homologous end joining repair (NHEJ), which repairs DSB in an error‐prone model,^[^ ^22^, ^23^ ^]^ homologous recombination (HR) repair is considered as the sole approach to accurately repair DSB in cells.^[^ ^24^ ^]^ It has been reported that defects of HR repair in fetal mouse oocytes might impair fertility.^[^ ^25^ ^]^ However, whether the defects of HR repair in trophoblast cells may lead to miscarriage is largely unknown and the potential mechanism is completely unexplored.

BRCA1 (breast cancer susceptibility gene 1) is a crucial protein that functions for HR repair.^[^ ^26^ ^]^ BRCA1 could suppress error‐prone NHEJ repair of DSB and promote accurate HR repair of DSB.^[^ ^27^ ^]^ Studies have shown that BRCA1 mutations lead to higher levels of DSB and are also associated with a variety of major diseases, such as epithelial ovarian cancer,^[^ ^28^ ^]^ breast cancer,^[^ ^29^ ^]^ and pancreatic cancer.^[^ ^30^ ^]^ Given the importance of BRCA1 for HR repair, it is of significance to explore what might regulate BRCA1 abundance in human trophoblast cells. On the other hand, phosphorylated H2AX (γ‐H2AX) might act as a platform to recruit components of the repair machinery such as BRCA1 to DSB foci to promote DSB repair.^[^ ^31^ ^]^ BRCA1 and γ‐H2AX form a physical complex to facilitate HR repair.^[^ ^32^ ^]^ The impaired interactions of BRCA1 with γ‐H2AX, which increase DSB levels, are associated with a variety of major diseases, such as cardiovascular diseases,^[^ ^33^ ^]^ ovarian aging,^[^ ^34^ ^]^ and cancers.^[^ ^35^ ^]^ However, it is still unclear whether and how BRCA1 might be recruited by γ‐H2AX to promote HR repair in human trophoblast cells.

LncRNAs (long non‐coding RNAs) have exhibited important regulatory functions in various biological processes.^[^ ^36^ ^]^ Increasing studies have shown that trophoblast cell functions are regulated by certain lncRNAs, such as lncRNA EPB41L4A‐AS1 which induces metabolic reprogramming in trophoblast cells and placenta tissue,^[^ ^37^ ^]^ lnc‐SLC4A1‐1 which epigenetically regulates unexplained recurrent pregnancy loss by activating CXCL8 and NF‐kB pathway,^[^ ^38^ ^]^ and lncRNA MEG8 which causes trophoblast dysfunctions and miscarriage by decreasing cell proliferation and invasion.^[^ ^39^ ^]^ In this group, we identified a number of novel lncRNAs, including lnc‐HZ01,^[^ ^12^ ^]^ lnc‐HZ03,^[^ ^7^ ^]^ lnc‐HZ04,^[^ ^10^ ^]^ lnc‐HZ05,^[^ ^11^ ^]^ lnc‐HZ08,^[^ ^8^ ^]^ lnc‐HZ09,^[^ ^15^ ^]^ and lnc‐HZ14,^[^ ^13^ ^]^ which regulate various trophoblast cell dysfunctions and the occurrence of miscarriage. Meanwhile, it has been reported that lncRNAs could also regulate HR repair.^[^ ^40^ ^]^ Loss of ATM‐regulated lncRNA, ANRIL, has been shown to impair HR repair in pancreatic cancer cells.^[^ ^41^ ^]^ In addition, a prostate‐specific lncRNA, PCAT‐1, regulates BRCA2 and controls HR repair in sporadic cancer cells.^[^ ^42^ ^]^ Lnc‐RI regulates the stability of RAD51 mRNA; and thereby, regulates HR repair of DSB.^[^ ^43^ ^]^ However, till now, lncRNAs that could simultaneously regulate HR repair in human trophoblast cells and the occurrence of miscarriage have never been reported. Moreover, whether lncRNAs might regulate BRCA1 expression and its recruitment to DSB foci in human trophoblast cells is also completely unknown and should be fully investigated.

The causes of ≈50% RM are still unexplained. Accumulating epidemiological investigations have revealed that exposure of pregnant women to environmental toxicants such as polycyclic aromatic hydrocarbons (PAHs) is closely associated with miscarriage.^[^ ^44^ ^]^ BaP (benzo(a)pyrene), a representative of PAHs, and its metabolite product, BPDE (benzo(a)pyren‐7,8‐dihydrodiol‐9,10‐epoxide), are typical environmental persistent organic pollutants and endocrine disruptors.^[^ ^45^ ^]^ Recently, we have found that BPDE induced trophoblast apoptosis, ferroptosis, and pyroptosis, and also inhibited trophoblast migration/invasion and proliferation.^[^ ^7^, ^8^, ^9^, ^10^, ^11^, ^12^, ^13^, ^46^ ^]^ In animal studies, we found that BaP exposure induced miscarriage of pregnant mice.^[^ ^7^, ^8^, ^9^, ^10^, ^11^, ^12^, ^13^, ^46^ ^]^ However, it is still unclear whether BPDE might impair HR repair in human trophoblast cells and whether this impairment might further induce miscarriage. Moreover, PAH exposure activates the expression of AhR (aryl hydrocarbon receptor).^[^ ^47^ ^]^ AhR may act as a transcription factor to perform the transcription of a wide range of genes that may exhibit the biological and toxicological effects of PAHs.^[^ ^48^ ^]^ It has been reported that AhR inhibited BRCA1 transcription in MCF7, UACC3199, and HCC38 BC cells.^[^ ^49^ ^]^ However, whether BPDE might regulate HR repair through AhR in human trophoblast cells is completely unknown and should be investigated.

Therefore, in this study, we expect to explore the correlation among BPDE exposure, defective HR repair in villous tissues (containing human trophoblast cells), and miscarriage. We want to identify a novel lncRNA that could regulate HR repair simultaneously in BPDE‐exposed trophoblast cells and RM villous tissues. Specially, we hope to discover regulatory roles of lncRNA in the regulation of BRCA1 expression and its recruitment to DSB foci in human trophoblast cells. Moreover, we also want to construct a mouse mode to discover how BaP/BPDE affects HR repair in mouse placental tissues and induces miscarriage. Based on this mechanism, we want to develop a miscarriage intervention strategy to alleviate miscarriage. Taken together, we hope to discover scientific and clinical insights in treatment against unexplained miscarriage.

## 2 Results

### 2.1 Defective HR Repair in Human Villous Tissues Was Associated With Miscarriage

To compare the differences and explore the pathogenesis of unexplained recurrent miscarriage (RM), in our previous studies,^[^ ^7^, ^8^, ^12^, ^13^, ^14^, ^15^, ^38^ ^]^ we have performed total transcriptome sequencing using villous tissue samples of unexplained RM group and their matched healthy control (HC) group. In this sequencing data, we found that BRCA1, a key protein that functions as a classical endogenous HR repair protein,^[^ ^50^ ^]^ was significantly lowly expressed in RM, relative to HC tissues (Figure S1A, Supporting Information). To confirm this, in this study, we newly collected RM and HC villous tissue samples (each *n* = 20), as the methods described previously.^[^ ^7^, ^8^, ^12^, ^13^, ^14^, ^15^, ^38^ ^]^ The RM group had experienced two or more consecutive unexplained spontaneous miscarriages; and the known causes, such as chromosomal abnormalities, hormonal abnormalities, and uterine deformation, had been excluded.^[^ ^7^, ^8^, ^12^, ^13^, ^14^, ^15^, ^38^ ^]^ Then, RT‐qPCR and Western blot analysis confirmed that the expression levels of BRCA1, as well as of another important HR repair protein, RAD51,^[^ ^51^, ^52^, ^53^ ^]^ were lower in RM versus in HC villous tissues (**Figure** **1**A–D). Meanwhile, the levels of dsDNA beak (DSB), as indicated by the protein levels of γ‐H2AX, were higher in RM versus HC villous tissues (Figure 1B,E). To explore whether BRCA1 might promote HR repair in human trophoblast cells, we overexpressed or silenced BRCA1 in Swan 71 or HTR‐8/SVneo cells (Figure S1B,C, Supporting Information) and found that BRCA1 overexpression promoted trophoblast cell HR repair (Figure 1F,G); whereas, BRCA1 knockdown suppressed HR repair (Figure 1F,G), agreeing with the results reported in other cell lines.^[^ ^54^ ^]^ As a result, the levels of DSB (as indicated by γ‐H2AX protein levels) were lower in BRCA1‐overexpressed trophoblast cells but higher in BRCA1‐silenced trophoblast cells (Figure 1H). Collectively, these results showed that HR repair, as indicated by lower levels of BRCA1 and RAD51 and higher levels of DSB, was suppressed in RM versus HC villous tissues and the defective HR repair in villous tissues was associated with miscarriage.

Figure 1A novel lnc‐HZ10 was up‐regulated in RM versus HC villous tissues and suppressed HR repair by down‐regulating BRCA1 in human trophoblast cells. A) RT‐qPCR analysis of BRCA1 mRNA levels in healthy control (HC) and unexplained recurrent miscarriage (RM) groups (each *n* = 20). B–E) Western blot analysis and the relative quantification of the protein levels of BRCA1, RAD51, and γ‐H2AX in RM and HC villous tissues (each *n* = 10), with β‐Tubulin as loading control. F,G) Flow cytometry analysis of the relative HR efficiency in Swan 71 (F) and HTR‐8/SVneo (G) cells with overexpression or knockdown of BRCA1. H) Western blot analysis and the relative quantification of BRCA1 and γ‐H2AX protein levels in Swan 71 or HTR‐8/SVneo cells with overexpression or knockdown of BRCA1, with β‐Tubulin as loading control. I) RT‐qPCR analysis of a novel lnc‐14627 (named as lnc‐HZ10 later) levels in HC and RM villous tissues (each *n* = 20). J) RNA FISH analysis of lnc‐HZ10 subcellular localization in Swan 71 cells and its relative quantification in nucleus and cytoplasm (scale bar, 50 µm). K,L) Pearson analysis of the correlation between the levels of BRCA1 protein (K) or γ‐H2AX protein (L) and the levels of lnc‐HZ10 in HC (blue, *n* = 10) and RM (red, *n* = 10) villous tissues. M) Flow cytometry analysis of the relative HR efficiency in Swan 71 cells with overexpression or knockdown of lnc‐HZ10. N) Comet assay analysis and the relative quantification of DNA in tails in Swan 71 cells with overexpression or knockdown of lnc‐HZ10, with H_2_O_2_ as positive control. The percentage of DNA in tail was quantified. O) Western blot analysis and the relative quantification of BRCA1, RAD51, and γ‐H2AX protein levels in trophoblast cells with overexpression or knockdown of lnc‐HZ10, with β‐Tubulin as loading control. P) Immunofluorescence image analysis and the relative quantification of BRCA1 and γ‐H2AX protein foci in Swan 71 cells with overexpression of lnc‐HZ10 (each *n* = 100). Scale bar = 50 µm. Q,R) Flow cytometry analysis of the relative HR efficiency in Swan 71 (Q) or HTR‐8/SVneo (R) cells with knockdown of BRCA1 or/and knockdown of lnc‐HZ10. Representative data in (B, H left, J left, N,O left, P left) represent three independent experiments. Data in (A, C–G, H right, I, J right, K–M, N right, O,P right, Q, R) show mean ± SD of three independent experiments. Unpaired Student's *t*‐test for (A, C–E, I); Kruskal–Wallis test for (F,G left, J right); Kruskal–Wallis test followed by Wilcoxon pairwise comparisons for (F–H right, M–O right, Q, R); one‐way ANOVA tests followed by Tukey's multiple comparisons test for (P right). Vector, empty vector of pcDNA3.1; OE‐BRCA1, overexpression of BRCA1; NC, negative control of siRNA; si‐BRCA1, knockdown of BRCA1; OE‐HZ10, overexpression of lnc‐HZ10; NC, negative control of siRNA; si‐HZ10, knockdown of lnc‐HZ10; HC, healthy control; *n*, the number of biologically independent samples; RM, recurrent miscarriage.

**Figure:**
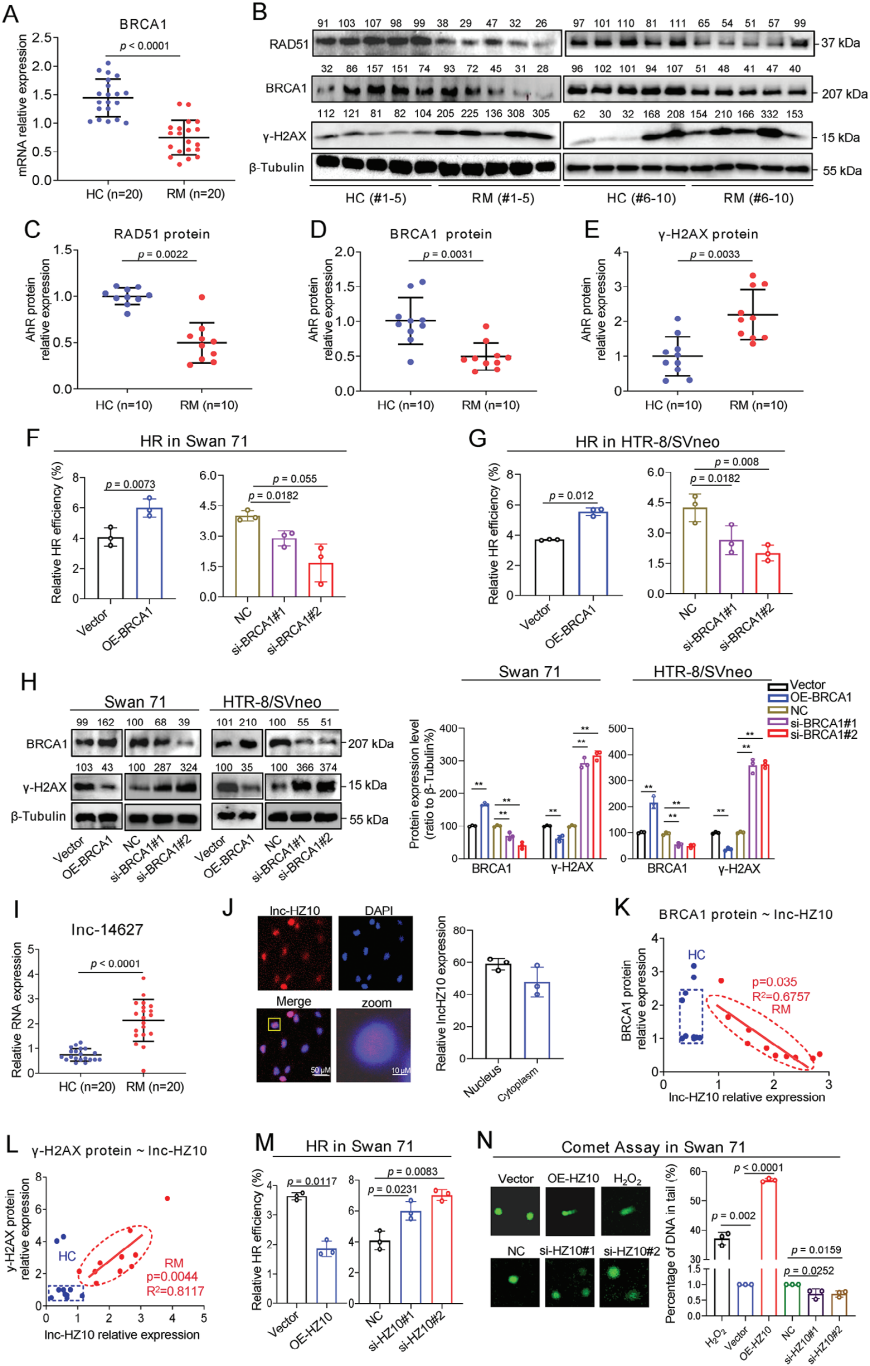

**Figure:**
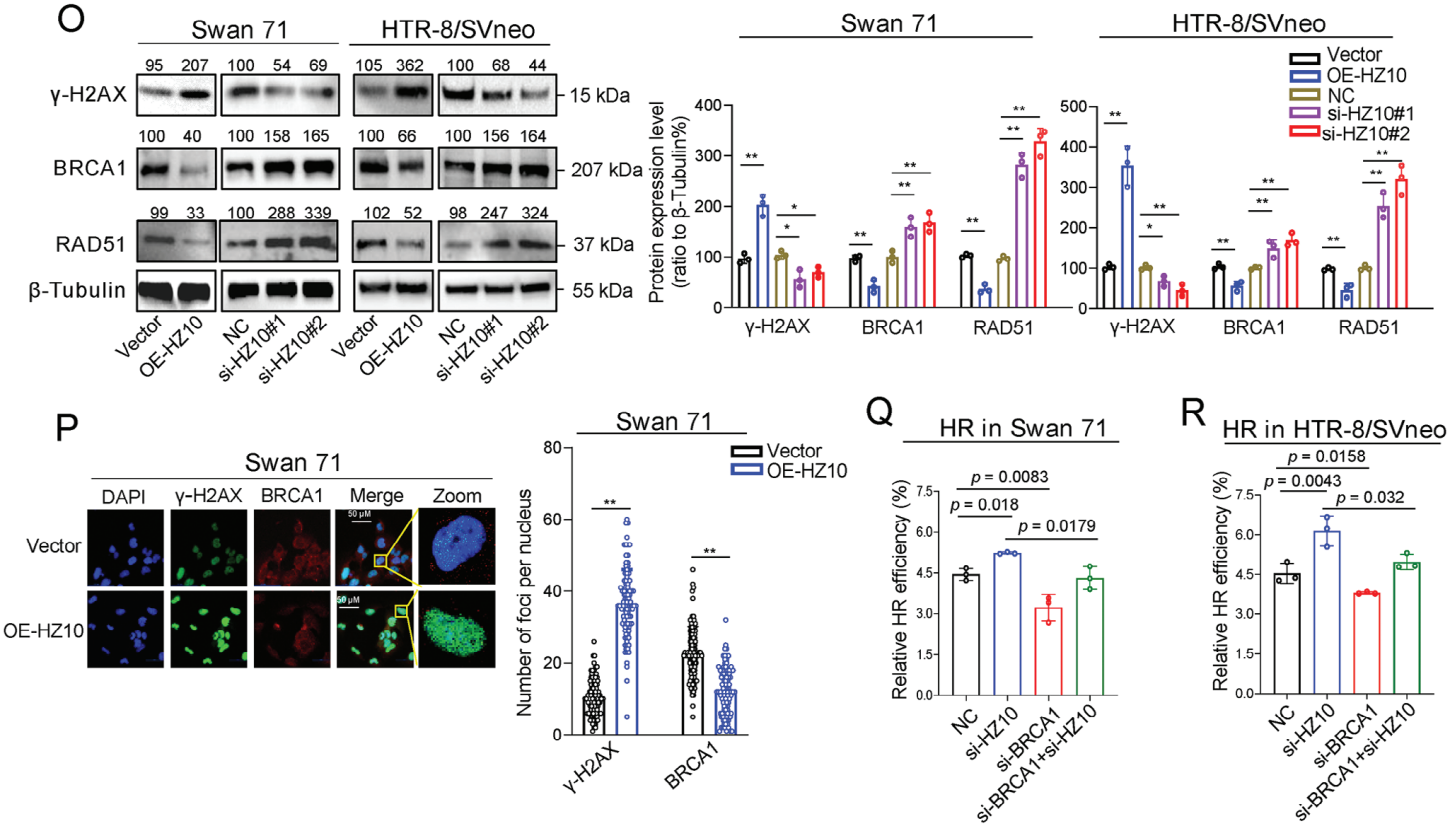

### 2.2 A Novel lnc‐HZ10 Was Highly Expressed in RM Versus HC Villous Tissues

In the sequencing data of RM versus HC villous tissues,^[^ ^7^, ^8^, ^12^, ^13^, ^14^, ^15^, ^38^ ^]^ we also identified a novel lnc‐14627 that was significantly highly expressed in RM versus HC villous tissues (Figure S1D, Supporting Information), which was further confirmed by RT‐qPCR analysis using the newly collected villous tissues (Figure 1I). Lnc‐14627 was identified as an antisense transcript with a full length of 2052 nt by RACE analysis (Figure S1E, Supporting Information) and located at chromosome 16 (chr 16: 3156683 – 3158734). Then, lnc‐14627 was also termed as lnc‐HZ10, and its full sequence had been submitted to NCBI with accession No. OK315569. The protein‐coding potential of lnc‐HZ10 was very weak, as predicted by the Coding Potential Assessment Tool (Figure S1F, Supporting Information) and NCBI ORF finder.^[^ ^55^ ^]^ In addition, lnc‐HZ10 had no conserved domains as predicted by the Conserved Domain Database and Pfam. Moreover, polyribosome binding assays also showed little interactions between lnc‐HZ10 and polyribosome (Figure S1G, Supporting Information). All these results indicated that lnc‐HZ10 might not encode a protein. FISH analysis showed that lnc‐HZ10 was distributed in both the nucleus and cytoplasm of Swan 71 cells (Figure 1J). Taken together, these results showed that a novel lnc‐HZ10 was highly expressed in RM versus HC villous tissues and its high expression might be associated with the occurrence of miscarriage.

### 2.3 Lnc‐HZ10 Suppressed HR Repair By Down‐Regulating BRCA1

Then, the regulatory functions of lnc‐HZ10 were explored. Pearson correlation analysis showed that the relative levels of lnc‐HZ10 were negatively correlated with those of BRCA1 protein (Figure 1K) and positively correlated with those of γ‐H2AX in RM groups (Figure 1L). These inspired us to explore whether lnc‐HZ10 might regulate BRCA1 expression and HR repair in trophoblast cells. To explore this, lnc‐HZ10 was overexpressed in Swan 71 cells (with empty vector cells as control) and its expression levels were verified by RT‐qPCR (Figure S1H (left), Supporting Information). Then, these cells were used for mRNA sequencing, generating 1301 up‐regulated and 1459 down‐regulated mRNAs with differences > twofold and *p* values < 0.05. KEGG analysis of these differentially expressed mRNAs showed that lnc‐HZ10 might regulate multiple biological processes, such as homogeneous recombination (HR), proliferation (MAPK and Hippo signaling pathway), migration/invasion (wnt signaling pathway), and apoptosis (p53 signaling pathway) (Figure S1I, Supporting Information). Among them, HR repair was significantly altered, indicating that lnc‐HZ10 might regulate HR repair in human trophoblast cells.

To validate this, lnc‐HZ10 was overexpressed or silenced in human trophoblast cells, and its expression levels were validated by RT‐qPCR analysis (Figure S1H,J, Supporting Information). HR assays (Figure 1M) and comet assays (Figure 1N; Figure S1K, Supporting Information) showed that lnc‐HZ10 overexpression suppressed HR repair and increased DSB levels; whereas, lnc‐HZ10 knockdown gave the opposite results. Moreover, overexpression of lnc‐HZ10 also up‐regulated the protein levels of γ‐H2AX in both trophoblast cells, and down‐regulated the mRNA and protein levels of BRCA1 and the protein levels of RAD51 (Figure S1L,M,O); whereas knockdown of lnc‐HZ10 down‐regulated the protein levels of γ‐H2AX and up‐regulated the mRNA and protein levels of BRCA1 and protein levels of RAD51 (Figure S1N,O, Supporting Information). After synchronization of Swan 71 cells in G2 phase, the effects of lnc‐HZ10 on HR repair efficiencies (Figure S1O, Supporting Information) and the protein levels of γ‐H2AX, BRCA1, and RAD51 were stronger, relative to those in the unsynchronized cells (Figure S1O vs Figure S1M, Supporting Information; and Figure S1P vs Figure S1O, Supporting Information). However, alteration of lnc‐HZ10 did not affect H2AX mRNA levels (Figure S1Q, Supporting Information). Nuclear/cytoplasmic fractionation assays showed that lnc‐HZ10 overexpression increased γ‐H2AX protein abundance and reduced BRCA1 protein abundance in the nucleus of trophoblast cells; in contrast, lnc‐HZ10 knockdown gave the opposite results (Figure S1R, Supporting Information). Confocal assays showed that lnc‐HZ10 overexpression increased γ‐H2AX protein abundance and reduced BRCA1 and RAD51 protein abundance in the nucleus of trophoblast cells (Figure 1P; Figure S1S–U, Supporting Information). These results confirmed that lnc‐HZ10 reduced BRCA1 and RAD51 abundance in trophoblast cell nucleus and suppressed HR repair. Co‐transfection assays further showed that the promotion of HR repair caused by lnc‐HZ10 knockdown was diminished by co‐silencing BRCA1 in human trophoblast cells (Figure 1Q,R). To explore whether lnc‐HZ10 might also affect the single‐strand break repair (SSBR) or NHEJ repair of DSB, we determined the protein levels of TopBP1 (the indicators of DNA repair),^[^ ^56^, ^57^, ^58^ ^]^ poly (ADP‐ribose) polymerase 1 (PARP1) and poly (ADP‐ribose) polymerase 2 (PARP2) (two indicators of SSBR),^[^ ^59^, ^60^, ^61^ ^]^ and 53BP1, Ku70, Ku80, and DNA‐PKcs (the indicators for NHEJ repair).^[^ ^22^, ^62^ ^]^ We found that that lnc‐HZ10 overexpression down‐regulated; whereas, lnc‐HZ10 knockdown up‐regulated the protein levels of TopBP1, PARP1, and PARP2 (Figure S1V, Supporting Information), indicating that lnc‐HZ10 might suppress the repair of SSB (single‐strand break) However, the levels of NHEJ repair proteins were almost unchanged in lnc‐HZ10‐overexpressed or ‐silenced trophoblast cells (Figure S1V, Supporting Information). Moreover, NHEJ repair assays also showed that alteration of lnc‐HZ10 did not affect NHEJ efficiencies (Figure S1W, Supporting Information). Therefore, lnc‐HZ10 little affected NHEJ efficiencies in human trophoblast cells. Therefore, we focused on the regulatory roles of lnc‐HZ10 in HR repair of DSB in this study. Taken together, these results indicated that lnc‐HZ10 suppressed HR repair by reducing BRCA1 protein abundance in the nucleus of trophoblast cells.

### 2.4 AhR Suppressed HR Repair By Suppressing BRCA1 mRNA Transcription

Subsequently, we explored how lnc‐HZ10 reduced BRCA1 abundance in trophoblast cells. It has been reported that AhR (aryl hydrocarbon receptor) could bind with the promoter (chr 17: 43125372–43125499) of BRCA1 and inhibit BRCA1 transcription in MCF7, UACC3199, and HCC38 BC cell lines.^[^ ^49^ ^]^ However, whether and how AhR affected BRCA1 transcription in human trophoblast cells was unknown. It was found that overexpression of AhR decreased; whereas, knockdown of AhR increased the mRNA and protein levels of BRCA1 in both trophoblast cells (Figure S2A–C, Supporting Information; **Figure**  **2**A). ChIP assays showed that this promoter region of BRCA1 could be enriched by AhR protein, which was further enhanced by overexpression of AhR (Figure 2B), indicating AhR suppressed BRCA1 transcription in trophoblast cells. Moreover, nuclear/cytoplasmic fractionation assays and confocal assays showed that overexpression of AhR reduced BRCA1 protein abundance in the nucleus of human trophoblast cells (Figure S2D, Supporting Information; Figure 2C). As for HR repair, overexpression of AhR decreased; whereas, knockdown of AhR increased HR repair efficiency in both trophoblast cells (Figure 2D; Figure S2E, Supporting Information). Collectively, these data indicated that AhR suppressed HR repair by suppressing BRCA1 mRNA transcription and its protein nuclear abundance in human trophoblast cells.

Figure 2Lnc‐HZ10 up‐regulated AhR protein levels by suppressing its CUL4B‐mediated ubquitination degradation; and thus, suppressed BRCA1 transcription. A) Western blot analysis and the relative quantification of AhR and BRCA1 protein levels in Swan 71 or HTR‐8/SVneo cells with overexpression or knockdown of AhR, with β‐Tubulin as loading control. B) PCR analysis of the levels of BRCA1 promoter region enriched by AhR protein in Swan 71 or HTR‐8/SVneo cells with overexpression of AhR in AhR ChIP assays, with IgG as negative control. C) Immunofluorescence image analysis of BRCA1 protein foci in Swan 71 or HTR‐8/SVneo cells with overexpression of AhR. Scale bar = 50 µm. D) Flow cytometry analysis of the relative HR repair efficiency in Swan 71 or HTR‐8/SVneo cells with overexpression of AhR. E) Western blot analysis and the relative quantification of AhR protein levels in Swan 71 or HTR‐8/SVneo cells with overexpression or knockdown of lnc‐HZ10, with β‐Tubulin as loading control. F) Protein stability of AhR in Swan 71 cells with overexpression or knockdown of lnc‐HZ10. G,H) Western blot analysis and the relative quantification of the levels of AhR‐Ub that were immunoprecipitated by AhR antibody in Swan 71 cells with overexpression or knockdown of I) lnc‐HZ10 or J) CUL4B in ubiquitination assays, with β‐Tubulin as loading control. Western blot analysis and the relative quantification of CUL4B protein levels in Swan 71 cells with overexpression or knockdown of lnc‐HZ10, with β‐Tubulin as loading control (I). The mRNA levels of CUL4B in Swan 71 cells with overexpression or knockdown of lnc‐HZ10 (J). K) CUL4B mRNA stability in Swan 71 cells with overexpression or knockdown of lnc‐HZ10. L) Western blot analysis and the relative quantification of AhR protein levels in Swan 71 or HTR‐8/SVneo cells with overexpression of lnc‐HZ10 or overexpression of CUL4B, with β‐Tubulin as loading control. M) Western blot analysis and the relative quantification of BRCA1 protein levels in Swan 71 or HTR‐8/SVneo cells with overexpression of lnc‐HZ10 or knockdown of AhR, with β‐Tubulin as loading control. N) PCR analysis of the levels of BRCA1 promoter region enriched by AhR protein in Swan 71 cells with overexpression of lnc‐HZ10 in AhR ChIP assays, with IgG as negative control. Representative data in (A left, C, E left, G–I left, L,M left) represent three independent experiments. Data in (A right, B–D, E right, F, G,I right, J, K, L,M right, N) show mean ± SD of three independent experiments. Kruskal–Wallis test for (D, F, J left); Kruskal–Wallis test followed by Wilcoxon pairwise comparisons for (A right, B, E right, G–J right, K, L,M right, N). OE‐AhR, overexpression of AhR; si‐AhR, knockdown of AhR; OE‐CUL4B, overexpression of CUL4B; and si‐CUL4B, knockdown of CUL4B.

**Figure:**
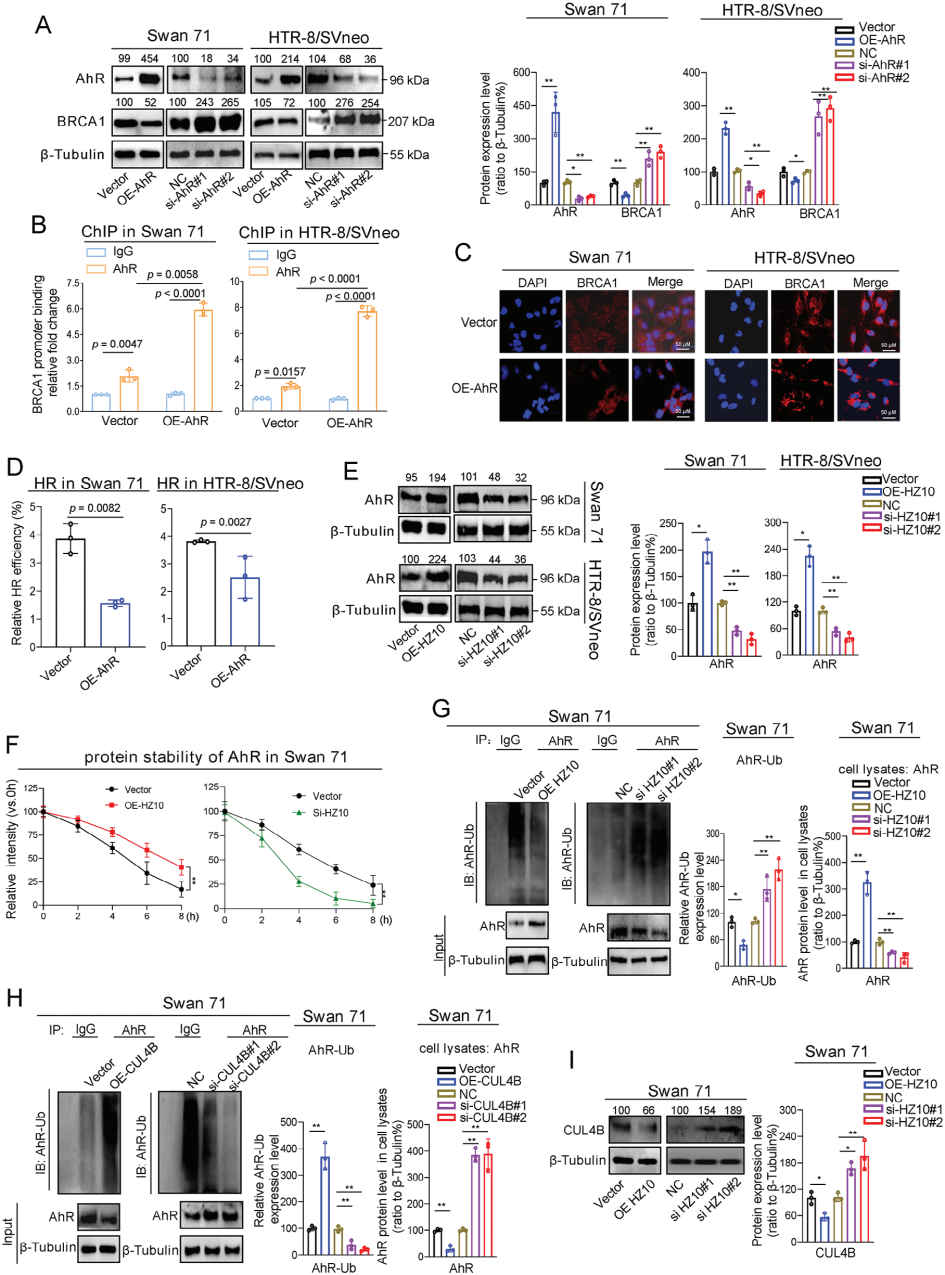

**Figure:**
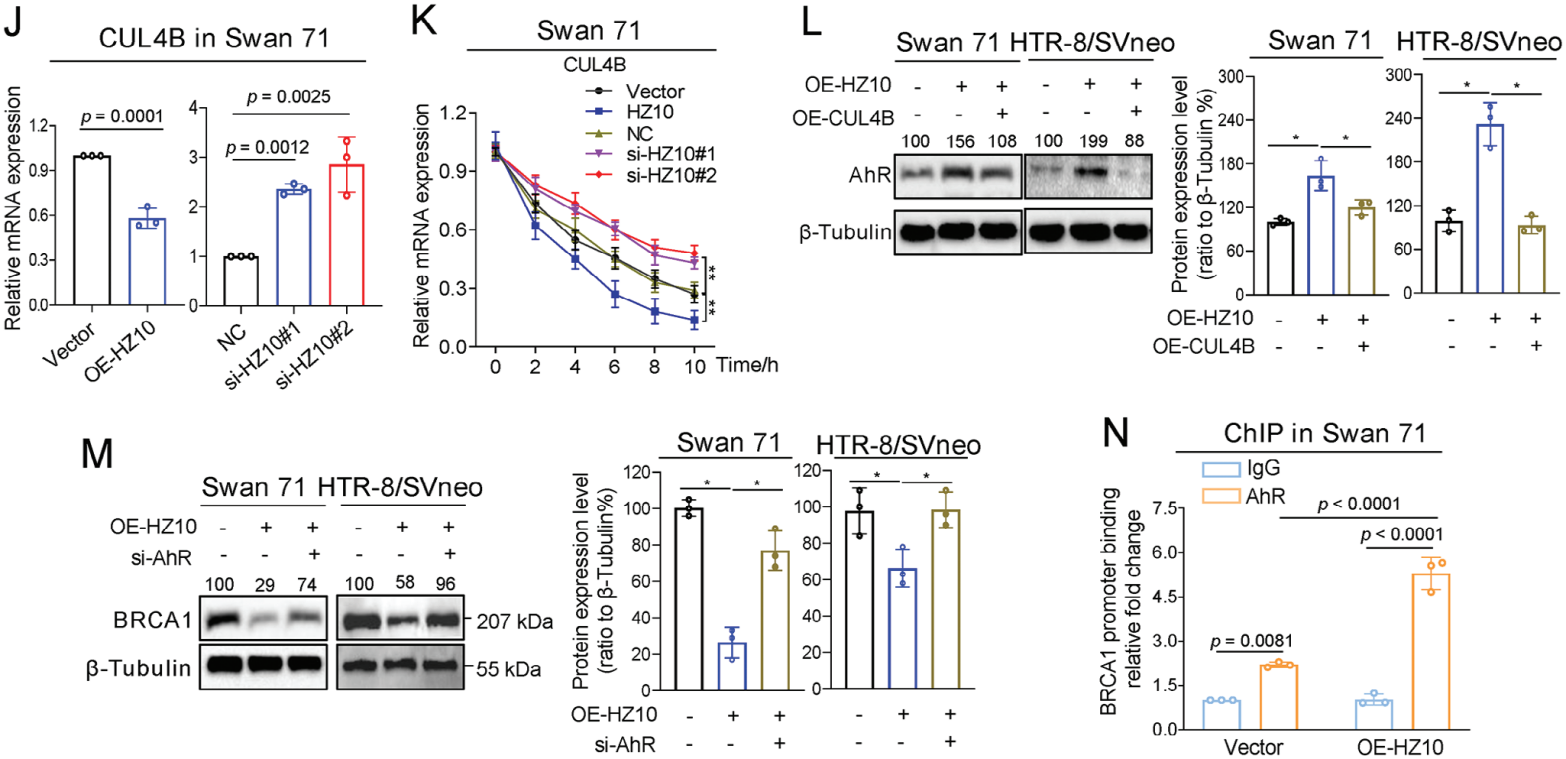

### 2.5 Lnc‐HZ10 Up‐Regulated AhR Levels by Suppressing its CUL4B‐Mediated Ubiquitination Degradation

Next, we further explored whether and how lnc‐HZ10 regulated AhR expression levels. First, we found that lnc‐HZ10 overexpression up‐regulated; whereas, lnc‐HZ10 knockdown down‐regulated AhR mRNA and protein levels in trophoblast cells and AhR protein levels in the nucleus of trophoblast cells (Figures S2F, S2G, and S2E, Supporting Information, respectively). Moreover, lnc‐HZ10 overexpression enhanced AhR protein stability; whereas, lnc‐HZ10 knockdown reduced AhR stability (Figures S2H and S2F, Supporting Information, respectively), indicating lnc‐HZ10 suppressed AhR protein degradation. It has been reported that protein poly‐ubiquitination is a key step to promote protein ubiquitin–proteasome degradation in eukaryotic cells.^[^ ^63^ ^]^ To validate this, we found that trophoblast cells treated with MG132 to block protein ubiquitin–proteasome degradation could significantly increase AhR protein accumulation (Figure S2I, Supporting Information). Moreover, lnc‐HZ10 overexpression decreased; whereas, lnc‐HZ10 silence increased the levels of the poly‐ubiquitinated AhR (AhR‐Ub) (Figure 2G), indicating that lnc‐HZ10 may inhibit ubiquitin–proteasome degradation of AhR in human trophoblast cells.

It was found that CUL4B was an E3 ubiquitin ligase of AhR in MEF cells.^[^ ^64^ ^]^ To explore this in human trophoblast cells, CUL4B was overexpressed or silenced, which was validated by RT‐qPCR and Western blot analysis (Figure S2J,K, Supporting Information) and the levels of AhR‐Ub and AhR were determined. CUL4B overexpression increased; whereas, CUL4B silence decreased the levels of AhR‐Ub in Swan71 cells (Figure 2H). As a result, CUL4B overexpression decreased; whereas, CUL4B silence increased AhR protein levels in Swan71 cell lysates (Figure 2H). Further, lnc‐HZ10 overexpression reduced; whereas, lnc‐HZ10 knockdown increased the protein levels of CUL4B in trophoblast cells (Figure 2I). To explore the potential reasons, RT‐qPCR and RNA stability assays showed that lnc‐HZ10 overexpression reduced CUL4B mRNA levels and weakened its mRNA stability; whereas, lnc‐HZ10 knockdown increased CUL4B mRNA levels and enhanced its mRNA stability in trophoblast cells (Figure 2J,K). Co‐transfection assays showed that the up‐regulation of AhR protein levels caused by lnc‐HZ10 overexpression was abolished by co‐overexpressing CUL4B (Figure 2L). These results suggested that lnc‐HZ10 down‐regulated CUL4B expression levels and suppressed CUL4B‐mediated poly‐ubiquitination of AhR; and thus, up‐regulated AhR protein levels in human trophoblast cells. Collectively, lnc‐HZ10 increased AhR protein abundance in the nucleus of trophoblast cells by suppressing CUL4B‐mediated ubiquitin–proteasome degradation of AhR.

Finally, we explored the joint effects of lnc‐HZ10 and AhR on the abundance of BRCA1 in trophoblast cells. Co‐transfection assays showed that down‐regulation of BRCA1 caused by lnc‐HZ10 overexpression was reversed by co‐silencing AhR in both trophoblast cells (Figure 2M). AhR ChIP assays showed that the levels of BRCA1 promoter region enriched by AhR were increased with overexpression of lnc‐HZ10 in both trophoblast cells (Figure 2N; Figure S2L, Supporting Information). Moreover, nuclear/cytoplasmic fractionation and confocal assays also displayed that the nucleus abundance of BRCA1 was reduced with overexpression of lnc‐HZ10, a reduction that was reversed by co‐silencing AhR in both trophoblast cells (Figure S2M–P, Supporting Information). Taken together, these results confirmed that lnc‐HZ10 reduced BRCA1 abundance in the nucleus of trophoblast cells by up‐regulating AhR levels; and thus, suppressing BRCA1 mRNA transcription.

### 2.6 Lnc‐HZ10 Impaired the Protein Interactions Between BRCA1 and γ‐H2AX

The recruitment of BRCA1 onto DSB foci is a crucial step for efficient HR repair.^[^ ^65^ ^]^ However, whether BRCA1 interacted with γ‐H2AX in human trophoblast cells was still unexplored. First, we found that BRCA1 and γ‐H2AX could be pulled down by each other in both Swan 71 and HTR‐8/SVneo cells by IP assays (Figure S3A,B, Supporting Information), indicating that BRCA1 interacted with γ‐H2AX. In order to reduce the influence of proteins in cell lysates, IP assays using a limited but identical amount of BRCA1 antibody showed that lnc‐HZ10 overexpression reduced; whereas, lnc‐HZ10 knockdown increased the levels of γ‐H2AX protein that was pulled down by BRCA1; although, γ‐H2AX protein levels in cell lysates were reversely changed (**Figure** **3**A,B). Similarly, IP assays using a limited but identical amount of γ‐H2AX antibody also showed that lnc‐HZ10 overexpression weakened their protein interactions (Figure S3C,D, Supporting Information); whereas, lnc‐HZ10 knockdown enhanced these interactions (Figure 3C,D) in both trophoblast cells. These results confirmed that lnc‐HZ10 impaired the protein interactions between γ‐H2AX and BRCA1. To further validate the roles of lnc‐HZ10, cell lysates were treated with RnaseA to completely degrade lnc‐HZ10 in cell lysates (Figure S3E, Supporting Information) with little affecting the protein levels of BRCA1 and γ‐H2AX in cell lysates. The degradation of lnc‐HZ10 resulted in more BRCA1 protein that was co‐IPed by γ‐H2AX after RNase A treatment in the co‐IP assays (Figure 3E,F), indicating that elimination of lnc‐HZ10 enhanced the protein interactions between BRCA1 and γ‐H2AX. Collectively, these results showed that lnc‐HZ10 impaired the binding between BRCA1 and γ‐H2AX in human trophoblast cells.

Figure 3Lnc‐HZ10 directly interacted with γ‐H2AX and impaired BRCA1 recruitment. A,B) Western blot analysis and the relative quantification of the levels of γ‐H2AX protein that were immunoprecipitated by BRCA1 in Swan 71 cells with overexpression or knockdown of lnc‐HZ10 in IP assays using a limited and identical amount of BRCA1 antibody, with IgG as negative control. C,D) Western blot analysis and the relative quantification of the levels of BRCA1 protein that were immunoprecipitated by γ‐H2AX in Swan 71 or HTR‐8/SVneo cells with knockdown of lnc‐HZ10 in IP assays using a limited and identical amount of γ‐H2AX antibody, with IgG as negative control. E,F) Western blot analysis and the relative quantification of the levels of BRCA1 protein immunoprecipitated by γ‐H2AX in lnc‐HZ10‐overexpressed Swan 71 or HTR‐8/SVneo cell lysates with Rnase A treatment in IP assays using a limited and identical amount of γ‐H2AX antibody, with IgG as negative control. G,H) RT‐qPCR analysis of the levels of lnc‐HZ10 enriched by γ‐H2AX or BRCA1 protein in Swan 71 cells with overexpression of lnc‐HZ10 in RIP assays, with IgG protein as negative control. I) Various segments of lnc‐HZ10. J) RT‐qPCR analysis of the levels of every lnc‐HZ10 segment (lnc‐HZ10‐S1‐S5) that was enriched by γ‐H2AX protein in HTR‐8/SVneo cells in RIP assays, with IgG protein as negative control. K) RT‐qPCR analysis of the levels of lnc‐HZ10 or lnc‐HZ10‐ΔS1 that was enriched by γ‐H2AX protein in Swan 71 cells in RIP assays, with IgG protein as negative control. L) Western blot analysis of the protein levels of γ‐H2AX and BRCA1 pulled down by biotin‐labeled lnc‐HZ10 or each of its five segments (lnc‐HZ10‐S1‐5) in Swan 71 or HTR‐8/SVneo cells in RNA pulldown assays, with beads as negative control. M–T) Western blot analysis and the relative quantification of the levels of BRCA1 protein that was immunoprecipitated by γ‐H2AX in Swan 71 (M–P) or HTR‐8/SVneo (Q–T) cells with overexpression of lnc‐HZ10, lnc‐HZ10‐S1, or lnc‐HZ10‐ΔS1 in IP assays using a limited and identical amount of γ‐H2AX antibody, with IgG as negative control. U) Nuclear/cytoplasmic fractionation assay analysis and the relative quantification of the levels of γ‐H2AX and BRCA1 protein in the nucleus and cytoplasm of Swan 71 cells with overexpression of lnc‐HZ10, lnc‐HZ10‐S1, or lnc‐HZ10‐ΔS1, with β‐Tubulin as cytoplasm marker and H3 as nucleus marker. V) Flow cytometry analysis of the relative HR repair efficiency in Swan 71 or HTR‐8/SVneo cells with overexpression of lnc‐HZ10, lnc‐HZ10‐S1, or lnc‐HZ10‐ΔS1. Representative data in (A–F left, L, M, Q, U left) represent three independent experiments. Data in (A–F right, G, H, J, K, N–P, R–T, U right, V) show mean ± SD of three independent experiments. Kruskal–Wallis test for (A right); Kruskal–Wallis test followed by Wilcoxon pairwise comparisons for (B–F right, G, H, J, K, N–P, R–T, U right, V). OE‐HZ10‐S1, overexpression of HZ10‐S1; si‐HZ10‐S1, knockdown of HZ10‐S1. OE‐HZ10‐ΔS1, overexpression of HZ10‐ΔS1; si‐HZ10‐ΔS1, knockdown of HZ10‐ΔS1.

**Figure:**
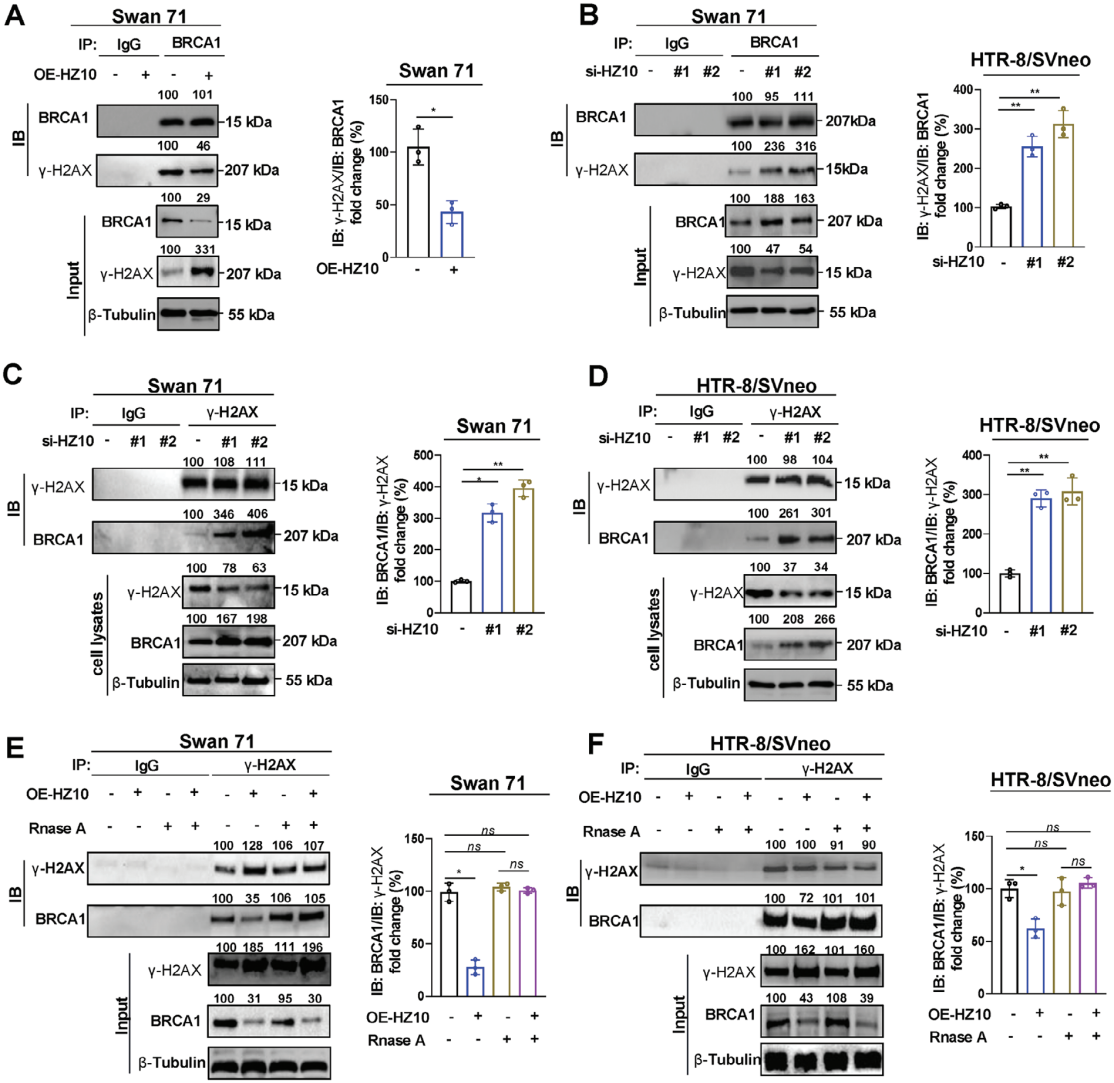

**Figure:**
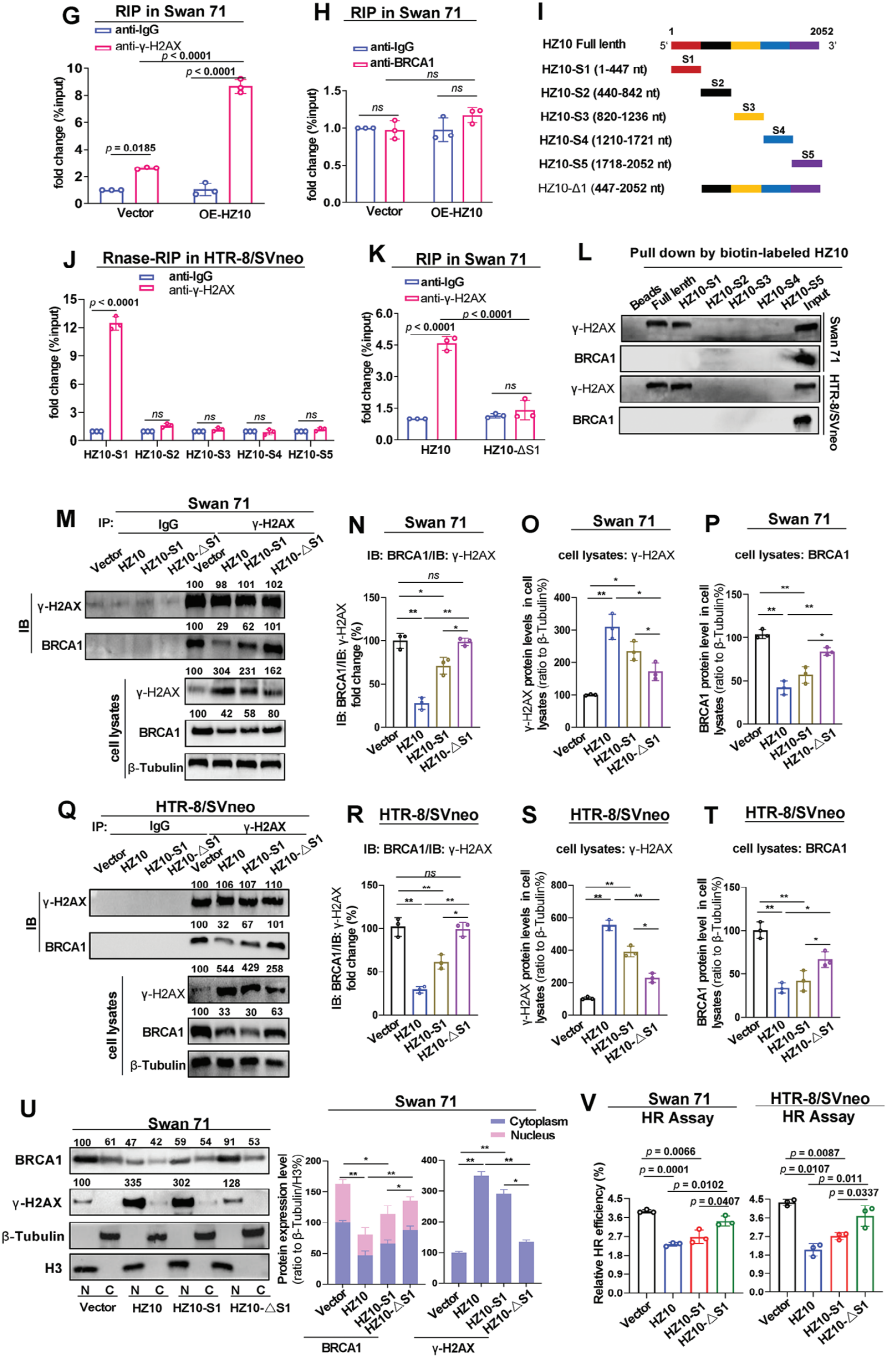

Subsequently, we explored whether lnc‐HZ10 might directly bind with BRCA1 or γ‐H2AX protein in trophoblast cells. RIP assays showed that lnc‐HZ10 could be pulled down by γ‐H2AX but not by BRCA1 (Figure 3G,H), indicating that lnc‐HZ10 might interact with only γ‐H2AX. To identify which segment of lnc‐HZ10 might interact with γ‐H2AX, lnc‐HZ10 was truncated into five segments (lnc‐HZ10‐S1–S5, Figure 3I) based on their secondary structures. Only the first segment (lnc‐HZ10‐S1, 1‐447 nt), but not others, could be pulled down by γ‐H2AX (Figure 3J). To explore the regulatory roles of this segment S1, we constructed lnc‐HZ10‐ΔS1 (lnc‐HZ01 deleting segment 1) (Figure 3I). RIP assays further showed that lnc‐HZ10, but not lnc‐HZ10‐ΔS1, could be pulled down by γ‐H2AX (Figure 3K), indicating that the first segment of lnc‐HZ10 interacted with γ‐H2AX. RNA pulldown assays using biotin‐labeled lnc‐HZ10 or its various segments further confirmed that γ‐H2AX could be pulled down by only the full length and the first segment of lnc‐HZ10 but not by other segments (Figure 3L). However, BRCA1 could not be pulled down by any segments of lnc‐HZ10 (Figure 3L). Taken together, only the first segment of lnc‐HZ10 interacted with γ‐H2AX.

Afterward, the effects of lnc‐HZ10‐S1 and lnc‐HZ10‐ΔS1 on the binding between BRCA1 and γ‐H2AX were investigated. Overexpression of lnc‐HZ10 or lnc‐HZ10‐S1, but not lnc‐HZ10‐ΔS1, suppressed the interactions between γ‐H2AX and BRCA1 (Figure 3M,N,Q,R). Further treatment of cell lysate with RnaseA could rescue the interactions between BRCA1 and γ‐H2AX, those which had been suppressed by overexpression of lnc‐HZ10‐S1 (Figure S3F, Supporting Information). However, overexpression of lnc‐HZ10‐ΔS1 or treatment with RnaseA had little effects on their protein interactions (Figure S3G, Supporting Information). Therefore, lnc‐HZ10‐S1 might primarily bind with γ‐H2AX; and thus, impair its interactions with BRCA1. Collectively, these results showed that the first segment of lnc‐HZ10 (lnc‐HZ10‐S1) suppressed the recruitment of BRCA1 by γ‐H2AX by directly interacting with γ‐H2AX in human trophoblast cells.

Subsequently, whether lnc‐HZ10‐S1 might regulate the protein levels of BRCA1 and γ‐H2AX in trophoblast cells and HR repair was studied. Overexpression of lnc‐HZ10 or lnc‐HZ10‐S1 obviously reduced BRCA1 protein levels and increased γ‐H2AX protein levels in trophoblast cell lysates (Figure 3M,O,P,Q,S,T). However, overexpression of lnc‐HZ10‐ΔS1 showed less effects on their protein levels compared with overexpression of lnc‐HZ10 or lnc‐HZ10‐S1 (Figure 3M,O,P,Q,S,T). Moreover, overexpression of lnc‐HZ10 or lnc‐HZ10‐S1 reduced BRCA1 nuclear abundance and increased γ‐H2AX nuclear abundance in trophoblast cells (Figure 3U; Figure S3H, Supporting Information). However, overexpression of lnc‐HZ10‐ΔS1 showed less effects on their abundance compared with overexpression of lnc‐HZ10 or lnc‐HZ10‐S1 (Figure 3U; Figure S3H, Supporting Information). As for HR repair, overexpression of lnc‐HZ10 or lnc‐HZ10‐S1 obviously suppressed HR repair; however, overexpression of lnc‐HZ10‐ΔS1 showed weaker suppression effect on HR repair in trophoblast cells (Figure 3V). Taken together, the first segment of lnc‐HZ10 (1‐447 nt) reduced BRCA1 nuclear abundance and suppressed its recruitment by γ‐H2AX; thus, suppressing HR repair in human trophoblast cells.

### 2.7 AhR Promoted lnc‐HZ10 Transcription

**Figure 4:**
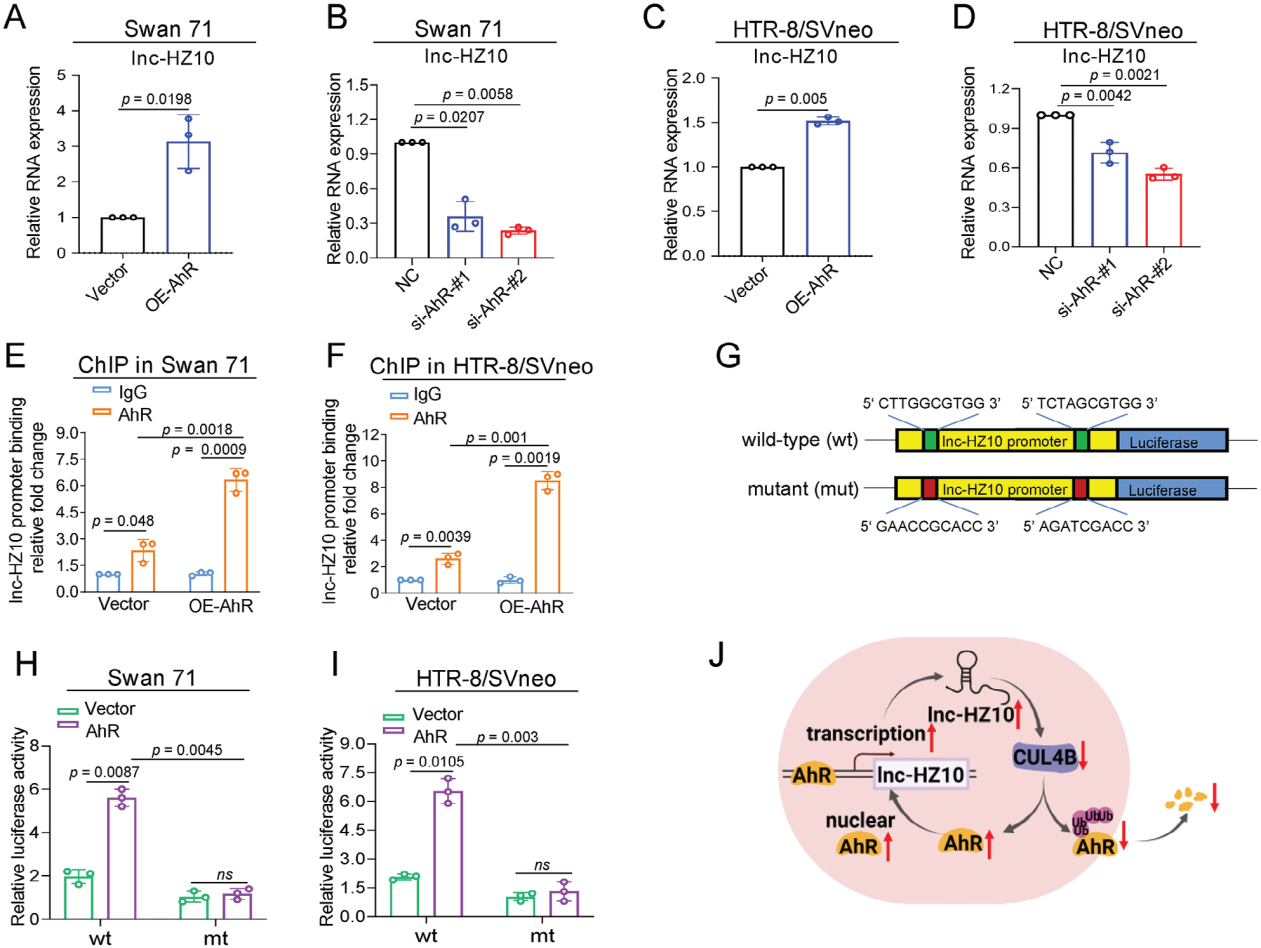
AhR promoted lnc‐HZ10 transpcription. A–D) RT‐qPCR analysis of lnc‐HZ10 levels in Swan 71 (A,B) or HTR‐8/SVneo (C,D) cells with overexpression (A,C) or knockdown (B,D) of AhR. E,F) PCR analysis of the levels of lnc‐HZ10 promoter region enriched by AhR protein in AhR‐overexpressed Swan 71 (E) or HTR‐8/SVneo (F) cells in AhR ChIP assays, with IgG as negative control. G) Wild‐type (wt) or mutant (mt) promoter sequence of lnc‐HZ10. H,I) Dual‐Luciferase reporter analysis of the transcription activity of AhR using wild‐type (wt) or mutant (mt) promoter sequence of lnc‐HZ10 in Swan 71 (H) or HTR‐8/SVneo (I) cells. J) The positive feedback‐loop mechanism. Data in (A–F, H,I) show mean ± SD of three independent experiments. Kruskal–Wallis test for (A,C); Kruskal–Wallis test followed by Wilcoxon pairwise comparisons for (B, D–F, H,I). wt, wild‐type lnc‐HZ10 promoter sequence; mt, mutant lnc‐HZ10 promoter sequence.

Subsequently, we explored how lnc‐HZ10 was regulated in trophoblast cells. Predicted by PROMO software, AhR might be a transcription factor of lnc‐HZ10. Experimentally, overexpression of AhR up‐regulated lnc‐HZ10 levels; whereas, knockdown of AhR down‐regulated lnc‐HZ10 levels in both trophoblast cells (**Figure** **4**A–D). ChIP assays showed that AhR could bind with the promoter region of lnc‐HZ10, which could be further enhanced by overexpression of AhR (Figure 4E,F). Dual‐luciferase reporter assays showed that AhR had transcription activity using wild‐type but not mutant sequence in the promoter region of lnc‐HZ10 (Figure 4G–I). All these results confirmed that AhR was a transcription factor of lnc‐HZ10 and promoted lnc‐HZ10 transcription in trophoblast cells.

### 2.8 Lnc‐HZ10 and AhR Formed a Positive Feedback Loop

Lnc‐HZ10 increased AhR abundance by suppressing its CUL4B‐mediated ubiquitination degradation. Meanwhile, AhR also acted as a transcriptional factor to promote lnc‐HZ10 transcription in trophoblast cells. Thus, both lnc‐HZ10 and AhR formed a positive auto‐regulatory feedback loop in human trophoblast cells (Figure 4J). This lnc‐HZ10/AhR loop further reduced nuclear BRCA1 abundance by inhibiting its transcription and also suppressed its recruitment to DSB foci by interacting with γ‐H2AX, both of which, impaired HR repair in human trophoblast cells.

### 2.9 Lnc‐HZ10/AhR Loop Was Highly Expressed in RM Villouse Tissues

**Figure 5:**
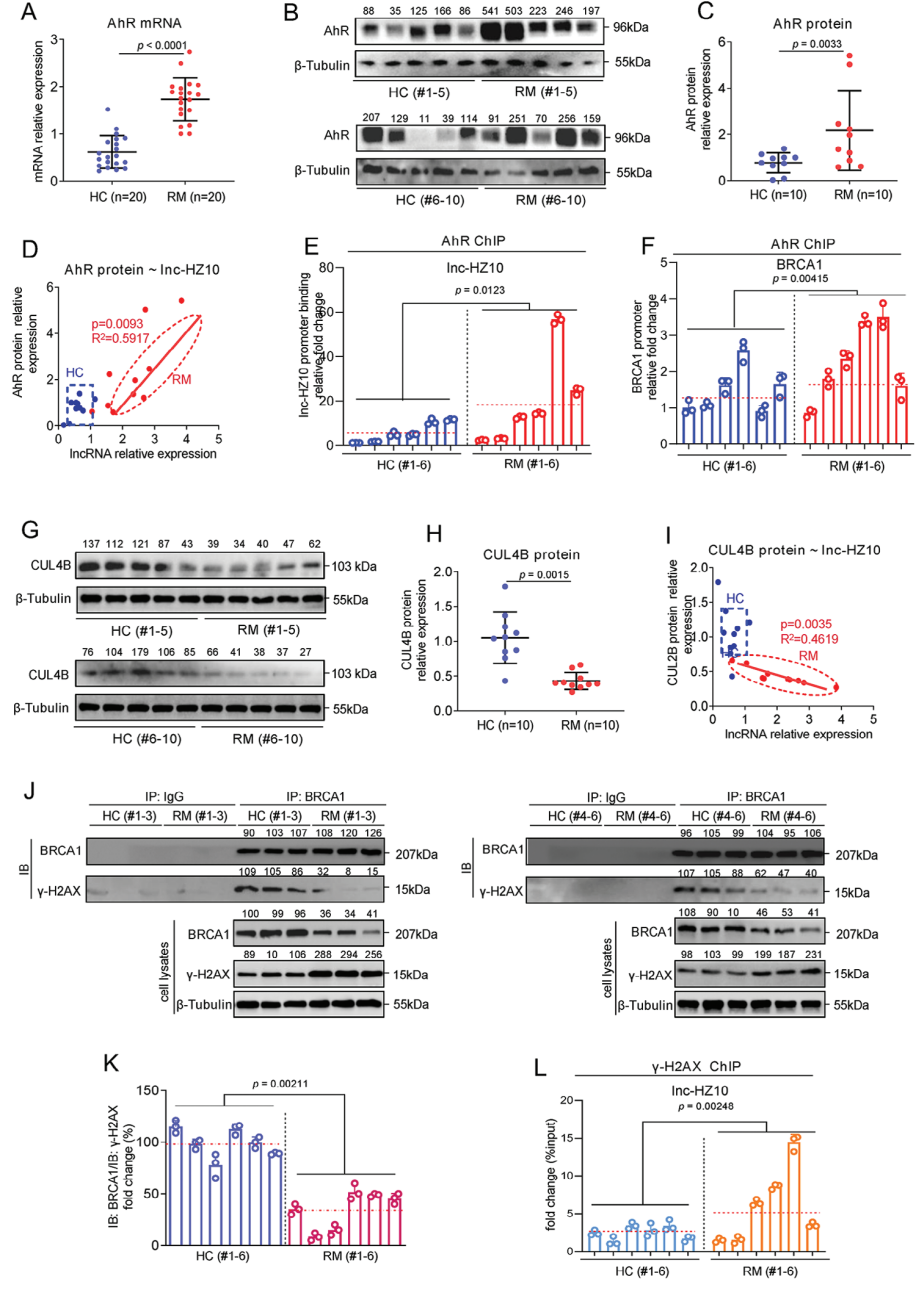
Lnc‐HZ10/AhR loop was highly expressed in RM versus HC villous tissues. A) RT‐qPCR analysis of AhR mRNA levels in healthy control (HC) and unexplained recurrent miscarriage (RM) villous tissues (each *n* = 20). B,C) Western blot analysis and the relative quantification of AhR protein levels in HC and RM villous tissues (each *n* = 10), with β‐Tubulin as loading control. D) The Pearson correlation analysis between the levels of AhR protein and those of lnc‐HZ10 in HC (blue) and RM (red) villous tissues (each *n*=12). E,F) PCR analysis of the levels of lnc‐HZ10 (E) or BRCA1 (F) promoter region enriched by AhR protein in HC and RM villous tissues (each *n* = 6) in AhR ChIP assays, with IgG as negative control. G,H) Western blot analysis and the relative quantification of CUL4B protein levels in HC and RM villous tissues (each *n* = 10), with β‐Tubulin as loading control. I) The Pearson correlation analysis of the levels of CUL4B protein and those of lnc‐HZ10 in HC (blue) and RM (red) villous tissues (each *n* = 12). J,K) Western blot analysis and the quantification of the levels of γ‐H2AX protein that were immunoprecipitated by BRCA1 in the HC and RM villous tissues (each *n* = 6) in RIP assays using a limited and identical amount of BRCA1 antibody, with IgG as negative control. L) RT‐qPCR analysis of the levels of lnc‐HZ10 that were enriched by γ‐H2AX protein in HC and RM villous tissues (each *n* = 6) in RIP assays, with IgG as negative control. Representative data in (B, G, J) represent three independent experiments. Data in (A, C–F, H, I, K, L) show mean ± SD of three independent experiments. Unpaired Student's *t*‐test for (A, C, H); one‐way ANOVA tests followed by Tukey's multiple comparisons test for (E,F, K,L).

To compare the expression levels of lnc‐HZ10/AhR loop and its downstream BRCA1 in RM and HC groups, their relative expression levels were determined in unexplained RM and HC villous tissues (each *n* = 20). The levels of lnc‐HZ10 were higher (Figure 1I), the mRNA and protein levels of AhR were higher (**Figure** **5**A–C), the mRNA and protein levels of BRCA1 and the protein levels of RAD51 were lower (Figure 1A–D), and the protein levels of γ‐H2AX were higher (Figure 1B,E) in RM versus HC groups. After normalization of their relative expression levels, the data points of lnc‐HZ10 and AhR protein levels in both groups were obviously separated (Figure 5D). The levels of lnc‐HZ10 were positively correlated with those of AhR or γ‐H2AX and negatively correlated with those of BRCA1 (Figures 5D and 1K,L), agreeing with the regulatory mechanisms in the cellular assays. Taken together, combined with the cellular results, we proposed that lnc‐HZ10 and AhR, both of which were highly expressed in RM versus HC villous tissues, might reduce BRCA1 expression levels and further suppress HR repair in RM villous tissues.

The potential mechanisms were further explored. For lnc‐HZ10, AhR ChIP assays showed that more promoter region of lnc‐HZ10 was enriched by AhR in RM versus HC villous tissues (Figure 5E), explaining higher levels of lnc‐HZ10 in RM versus HC group. For BRCA1, similarly, AhR ChIP assays showed that more BRCA1 promoter region was enriched by AhR in RM versus HC groups (Figure 5F), corresponding to lower expression levels of BRCA1 in RM versus HC group. Meanwhile, the protein levels of CUL4B were lower in RM versus HC villous tissues (Figure 5G,H). Pearson correlation analysis showed that the levels of lnc‐HZ10 were negatively correlated with those of CUL4B protein in RM villous tissues (Figure 5I), suggesting that lnc‐HZ10 might down‐regulate CUL4B expression levels in RM villous tissues. IP assays with a limited but identical amount of BRCA1 antibody showed that less γ‐H2AX was pulled down by BRCA1 in RM versus HC villous tissues (Figure 5J,K), indicating that less BRCA1 and γ‐H2AX protein complexes were formed in RM versus HC villous tissues. RIP assays showed that more lnc‐HZ10 was pulled down by γ‐H2AX in RM versus HC groups (Figure 5L). Taken together, combined with the cellular results, we proposed that the highly expressed AhR might promote lnc‐HZ10 transcription but suppress BRCA1 expression in RM villous tissues; and the up‐regulated lnc‐HZ10 might further suppress the recruitment of BRCA1 to DSB foci by interacting with γ‐H2AX in RM villous tissues.

### 2.10 BPDE Exposure Suppressed HR Repair By Up‐Regulating lnc‐HZ10/AhR Loop in Trophoblast Cells

Subsequently, we explored what might up‐regulate lnc‐HZ10 and AhR levels and suppress HR repair in RM versus HC villous tissues. Moreover, due to ethical and experimental limitations, it is impossible to directly evaluate the occurrence of miscarriage by weakening HR repair in human villous tissues. In our recent studies, we have found that BPDE exposure resulted in various dysfunctions of human trophoblast cells.^[^ ^7^, ^8^, ^9^, ^10^, ^11^, ^12^, ^13^ ^]^ Therefore, we constructed a BPDE‐exposed human trophoblast cell model. If the change trends of lnc‐HZ10, AhR, BRCA1, γ‐H2AX, and HR repair were similar in both BPDE‐exposed trophoblast cells and RM villous tissues, we might have built a bridge to connect BPDE exposure, defective HR repair in trophoblast cells, and the occurrence of recurrent miscarriage.

**Figure 6:**
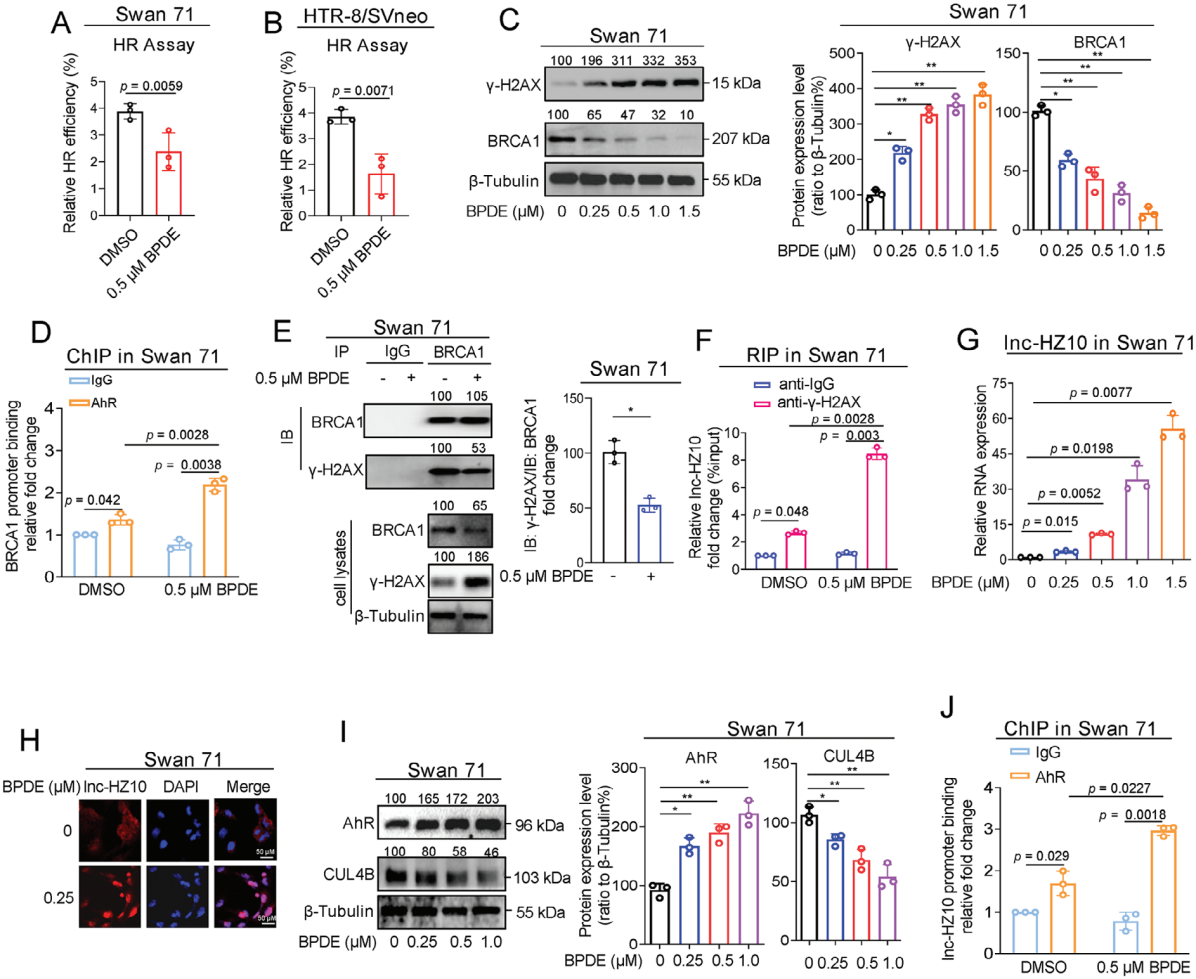
BPDE exposure suppressed HR repair by up‐regulating lnc‐HZ10/AhR loop in human trophoblast cells. A,B) Flow cytometry analysis of the relative HR repair efficiency in 0 or 0.5 µm BPDE‐exposed Swan 71 (A) or HTR‐8/SVneo (B) cells. C) Western blot analysis and the relative quantification of γ‐H2AX and BRCA1 protein levels in 0, 0.25, 0.5, 1.0, or 1.5 µm BPDE‐exposed Swan 71 cells, with β‐Tubulin as loading control. D) PCR analysis of the levels of BRCA1 promoter region enriched by AhR protein in 0 or 0.5 µm BPDE‐exposed Swan 71 cells in AhR ChIP assays, with IgG as negative control. E) Western blot analysis and the relative quantification of the levels of γ‐H2AX protein that were immunoprecipitated by BRCA1 in 0 or 0.5 µm BPDE‐exposed Swan 71 cells in IP assays using a limited and identical amount of BRCA1 antibody, with IgG as negative control. F) RT‐qPCR analysis of the levels of lnc‐HZ10 that were enriched by γ‐H2AX protein in 0 or 0.5 µm BPDE‐exposed Swan 71 cells in RIP assays, with IgG protein as negative control. G) RT‐qPCR analysis of lnc‐HZ10 levels in 0, 0.25, 0.5, 1.0, or 1.5 µm BPDE‐exposed Swan 71 cells. H) RNA FISH analysis of lnc‐HZ10 subcellular localization in 0 or 0.25 BPDE‐exposed Swan 71 cells (scale bar, 50 µm). I) Western blot analysis and the relative quantification of AhR and CUL4B protein levels in 0, 0.25, 0.5, or 1.0 µm BPDE‐exposed Swan 71 cells, with β‐Tubulin as loading control. J) PCR analysis of the levels of lnc‐HZ10 promoter region enriched by AhR protein in 0 or 0.5 µm BPDE‐exposed Swan 71 cells in AhR ChIP assays, with IgG as negative control. Representative data in (C left, E left, H, I left) represent three independent experiments. Data in (A,B, C right, D,E right, F,G, I right, J) show mean ± SD of three independent experiments. Kruskal–Wallis test for (A, B, E right); Kruskal–Wallis test followed by Wilcoxon pairwise comparisons for (C right, D, F,G, I right, J).

First, BPDE exposure reduced HR repair efficiency (**Figure** **6**A,B), increased γ‐H2AX protein levels (Figure 6C), and reduced BRCA1 levels (Figure 6C), suggesting that BPDE exposure suppressed HR repair in trophoblast cells. Then, the underlying mechanisms of HR repair were explored. In BPDE‐exposed trophoblast cells, AhR ChIP assays showed that more BRCA1 promoter region was enriched by AhR (Figure 6D), explaining that BRCA1 transcription was suppressed in BPDE‐exposed cells. IP assays using a limited but identical amount of BRCA1 antibody showed that less γ‐H2AX was pulled down by BRCA1 in BPDE‐exposed cells (Figure 6E). Moreover, RIP assays showed that more lnc‐HZ10 was enriched by γ‐H2AX in BPDE‐exposed cells (Figure 6F). Taken together, BPDE exposure reduced BRCA1 expression levels and impaired its recruitment by γ‐H2AX, both of which suppressed HR repair in BPDE‐exposed trophoblast cells.

Then, lnc‐HZ10 expression levels were also analyzed. In BPDE‐exposed trophoblast cells, lnc‐HZ10 was highly expressed as determined by RT‐qPCR (Figure 6G) and FISH analysis (Figure 6H). The protein levels of AhR were up‐regulated and those of CUL4B were down‐regulated in BPDE‐exposed trophoblast cells (Figure 6I), indicating that BPDE (an aromatic hydrocarbon) exposure activated the expression of AhR (an aromatic hydrocarbon receptor), at least by suppressing CUL4B‐mediated ubiquitin–proteasome degradation of AhR. Moreover, AhR ChIP assays showed that more promoter region of lnc‐HZ10 was enriched by AhR in BPDE‐exposed trophoblast cells (Figure 6J), explaining higher expression levels of lnc‐HZ10 in BPDE‐exposed trophoblast cells. Therefore, BPDE exposure triggered and up‐regulated AhR/lnc‐HZ10 feedback loop in BPDE‐exposed trophoblast cells. Taken together, the up‐regulated AhR suppressed BRCA1 transcription, and the up‐regulated lnc‐HZ10 also impaired the protein interactions between BRCA1 and γ‐H2AX by directly interacting with γ‐H2AX, both of which suppressed HR repair in BPDE‐exposed trophoblast cells.

### 2.11 Defective HR Repair in Mouse Placenta Induced Miscarriage in BaP‐Exposed Mouse Model

In order to directly evaluate the causality from BaP exposure to placental tissue dysfunctions, and then, to miscarriage, we constructed a mouse model in which pregnant mice were exposed with BaP by oral gavage to induce miscarriage, as described previously.^[^ ^7^, ^8^, ^12^, ^13^, ^14^, ^15^, ^38^ ^]^ The miscarriage rates were increased with increasing BaP concentrations (**Figure** **7**A). UCSC‐BLAT analysis revealed that AhR, BRCA1, and H2AX were conserved in genome sequence among human, rhesus, mouse, dog, and elephant; but, lnc‐HZ10 was conserved only in human and Rhesus genomes (Figure S4A, Supporting Information). There were only a 120‐nt fragment and a 75‐nt fragment in mouse gene that were similar to the partial sequence of lnc‐HZ10 (total 2052 nt, Table S8, Supporting Information). However, the expression levels of these two fragments were undetectable in mouse placental tissues (Figure S4B, Supporting Information). Proteins and mRNAs were extracted from a random placenta in each mouse uterus. The mRNA and protein levels of murine Brca1 were decreased; whereas, the protein levels of Ahr and γ‐H2ax were increased in BaP‐exposed mouse placenta, relative to control placenta (Figure S4C, Supporting Information; Figure 7B‐E), a tendency similar to that found in BPDE‐exposed human trophoblast cells and in RM villous tissues. However, the mRNA levels of Ahr and H2ax were almost unchanged in BaP‐exposed mouse placenta (Figure S4D,E, Supporting Information).

Figure 7Defective HR repair in mouse placenta induced miscarriage in BaP‐exposed mouse model. A) Miscarriage rates in 0, 0.05, or 0.2 mg kg^−1^ BaP‐exposed mice (each *n* = 6). B–E) Western blot analysis and the relative quantification of the protein levels of murine Ahr, Brca1, and γ‐H2ax in placental tissues of 0, 0.05, or 0.2 (each *n* = 6) mg kg^−1^ BaP‐exposed mice, with β‐Tubulin as loading control. F) PCR analysis of the levels of murine Brca1 promoter region enriched by Ahr protein in placental tissues of BaP‐exposed mice (each *n* = 6) in Ahr ChIP assays, with IgG protein as negative control. G) Western blot analysis and the relative quantification of the levels of BRCA1 protein that were immunoprecipitated by γ‐H2AX in placental tissues of BaP‐exposed mice (each *n* = 6) in IP assays using a limited and identical amount of γ‐H2AX antibody, with IgG as negative control. H) Schematic diagram of an intervention model using BaP‐exposed mice. I) Representative morphology of uterus in control mice, BaP‐exposed mice, BaP + AS‐NC‐treated mice, and BaP + AS‐Ahr‐treated mice. J) The averages of miscarriage rates in these four groups of mice (each *n* = 6). K–N) Western blot analysis and the relative quantification of the protein levels of murine Ahr, γ‐H2ax, and Brca1 in placental tissues of AS‐Ahr‐treated and/or BaP‐exposed mice (each *n* = 6), with β‐Tubulin as loading control. O) Pearson correlation analysis of the protein levels of Brca1 and Ahr in AS‐Ahr‐treated group (red) and the BaP control group (blue) (each *n* = 6). P) Western blot analysis and the relative quantification of the levels of BRCA1 protein that was pulled down by γ‐H2ax in placental tissues of AS‐Ahr‐treated and/or BaP‐exposed mice (each *n* = 6) in IP assays using an excessive and identical amount of γ‐H2ax antibody, with IgG as negative control. Representative data in (B,G,I,K,P) represent three independent experiments. Data in (A,C–F,G right,J,L–O,P right) show mean ± SD of three independent experiments. Unpaired Student's *t*‐test for (L–N); Kruskal–Wallis test followed by Wilcoxon pairwise comparisons for (A,J); one‐way ANOVA tests followed by Tukey's multiple comparisons test for (C–F,G right,P right).

**Figure:**
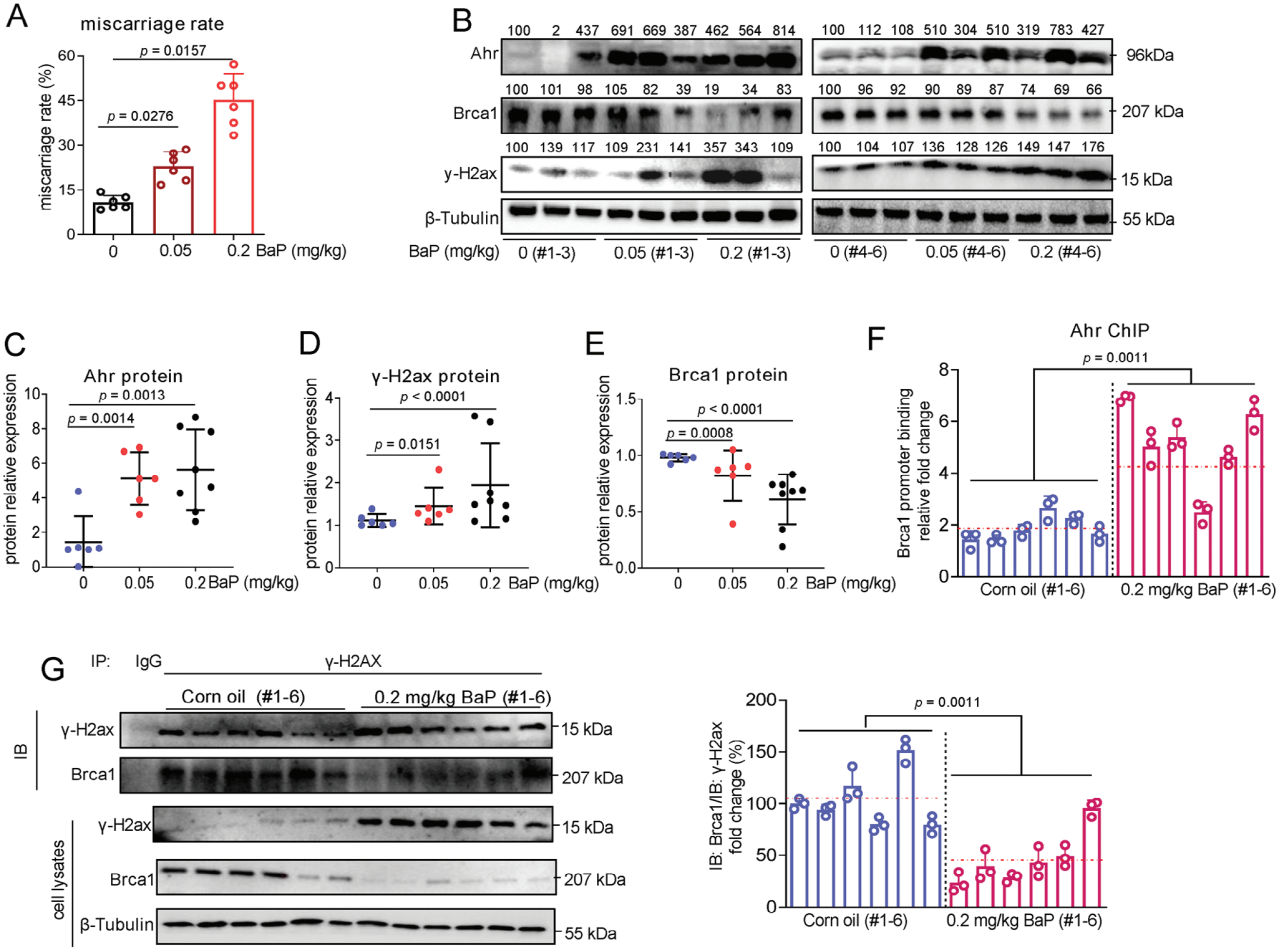

**Figure:**
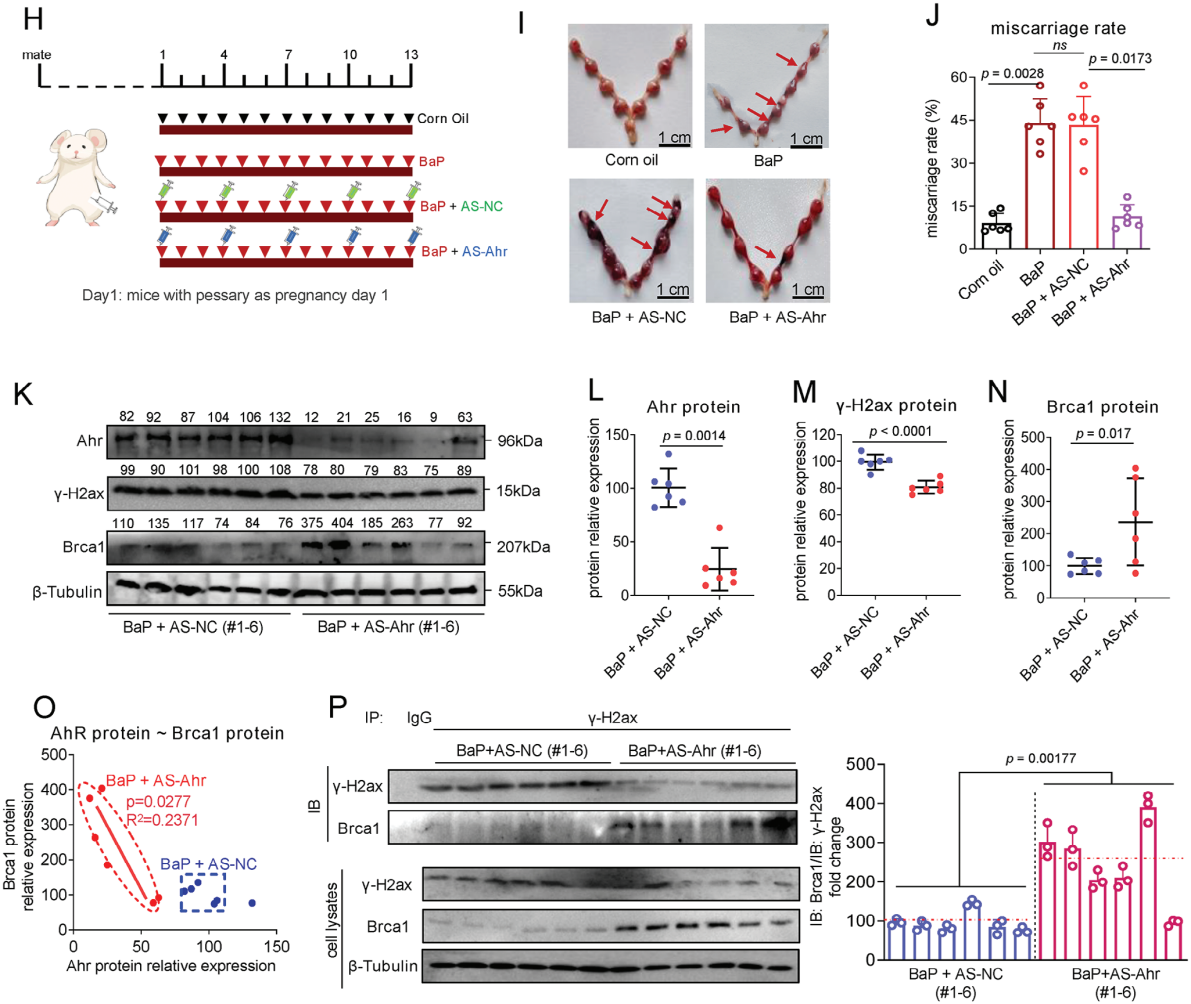

Subsequently, the underlying mechanisms were further explored. Predicted by PROMO, murine Ahr might bind with the promoter region of Brca1. Ahr ChIP assays showed that more Brca1 promoter region was enriched by Ahr in BaP‐exposed placental tissues (Figure 7F), explaining that Ahr‐suppressed Brca1 transcription was enhanced, which reduced Brca1 abundance in BaP‐exposed mouse placenta. Moreover, IP assays using a limited but identical amount of γ‐H2ax antibody showed that less levels of Brac1 were pulled down by γ‐H2ax in BaP‐exposed placenta (Figure 7G), implying that the recruitment of Brac1 by γ‐H2ax was reduced after BaP exposure. These data supported that the expression levels of Brac1 were lower, and less Brac1 protein was recruited to DSB foci by γ‐H2ax in BaP‐exposed group. Collectively, combined with the cellular results, we proposed that BaP exposure activated Ahr expression, suppressed Brac1 transcription; and thus, reduced Brac1 abundance and its recruitment, which further suppressed HR repair in BaP‐exposed mouse placenta.

### 2.12 Knockdown of Ahr Recovered HR Repair and Alleviated Miscarriage in BaP‐Exposed Mouse Miscarriage Model

Having known the mechanism underlying miscarriage in BaP‐exposed mouse model; next, we explored how to alleviate miscarriage. As lnc‐HZ10 homology was not observed in mouse genome, we chose mouse Ahr in the miscarriage intervention assays. For this aim, we constructed a miscarriage intervention model in which antisense oligonucleotide of Ahr (AS‐Ahr), with AS‐NC as control, was intraperitoneally injected into BaP‐exposed pregnant mice once per 3 days (Figure 7H). In the control group, BaP exposure led to embryo resorption; and thus, increased miscarriage rates; however, they were reduced in the AS‐Ahr‐treated group (Figure 7I,J). Analysis of placental tissues showed that the protein levels of Ahr and γ‐H2ax were reduced and those of Brca1 were increased in the AS‐Ahr‐treated group compared to those in the BaP control group (Figure 7K‐N). Pearson correlation analysis showed that the protein levels of Brca1 and Ahr were negatively correlated in the AS‐Ahr‐treated group (Figure 7O), suggesting that Ahr might negatively regulate Brca1 expression. IP assays using excessive and identical amount of γ‐H2ax antibody showed that less γ‐H2ax was enriched by γ‐H2ax antibody, but, more Brac1 was pulled down by γ‐H2ax in AS‐Ahr‐treated groups relative to those in the BaP control group (Figure 7P). Collectively, knockdown of Ahr could efficiently recover HR repair in placental tissues and alleviate miscarriage rates in the BaP‐exposed mouse model. These results also indicated that knockdown of AhR might act as a promising therapeutic strategy to prevent BaP‐induced miscarriage.

## 3 Discussion

### 3.1 Lnc‐HZ10 Regulates HR Repair

Studies have shown that several lncRNAs regulate HR repair. For example, lncRNA TERRA promotes the formation of DNA‐RNA hybrids on telomeres,^[^ ^66^ ^]^ lncRNA NORAD interacts with the proteins of DNA replication or repair and translocates them into nucleus,^[^ ^67^ ^]^ lncRNA ANRIL changes cell cycle checkpoints,^[^ ^41^ ^]^ lncRNA PCAT1 represses post‐transcription of BRCA2 in prostate tissue,^[^ ^68^ ^]^ and lnc‐RI regulates RAD51 mRNA stability,^[^ ^43^ ^]^ all of which regulate HR repair. Recently, we have found several novel lncRNAs that regulate trophoblast cell dysfunctions.^[^ ^7^, ^8^, ^12^, ^13^, ^14^, ^15^, ^38^ ^]^ However, till now, no lncRNAs were reported to regulate HR repair in human trophoblast cells. In our recent total transcriptome sequencing data, lnc‐HZ10 was one of the most highly expressed novel lncRNAs in RM versus HC villous tissues. In sequencing data of lnc‐HZ10‐overexpressed trophoblast cells, HR repair was one of the most significantly regulated cell phenotypes. Therefore, in this study, we selected lnc‐HZ10 and HR repair and explored the regulatory stream from BPDE exposure to high expression of lnc‐HZ10, and then, to HR repair. We found that lnc‐HZ10 suppressed HR repair in human trophoblast cells by reducing BRCA1 nuclear abundance and impairing BRCA1 recruitment to DSB foci, discovering a new approach to epigenetically regulate HR repair.

### 3.2 Regulatory Mechanism

**Figure 8:**
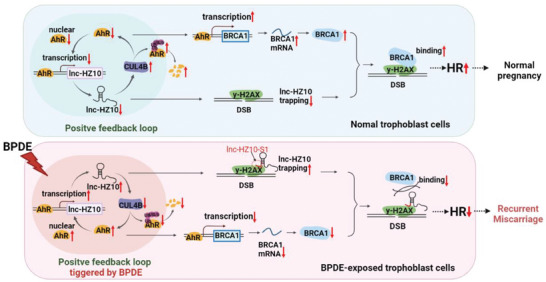
The proposed mechanism. AhR acted as a transcription factor to promote lnc‐HZ10 transcription. Meanwhile, lnc‐HZ10 up‐regulated AhR expression levels by suppressing its CUL4B‐mediated ubiquitination degradation. Thus, lnc‐HZ10 and AhR formed a positive feedback loop. Further, AhR suppressed BRCA1 transcription and reduced its abundance. The first fragment of lnc‐HZ10 (lnc‐HZ10‐S1) interacted with γ‐H2AX and impaired the recruitment of BRCA1 by γ‐H2AX. In normal trophoblast cells, AhR/lnc‐HZ10 feedback loop was lowly expressed. As a result, BRCA1 was highly expressed and HR repair was highly activated, giving normal pregnancy. Upon BPDE exposure, AhR was activated, and subsequently, triggered this feedback loop, which suppressed HR repair and possibly induced miscarriage.

In this work, we correlated BPDE exposure, defective HR repair in trophoblast cells, and the occurrence of miscarriage. A novel lnc‐HZ10 was identified, which was highly expressed in RM versus HC villous tissues and in BPDE‐exposed trophoblast cells. The regulation mechanisms were proposed (**Figure** **8**). AhR, a transcription factor of lnc‐HZ10, promoted lnc‐HZ10 transcription. Meanwhile, lnc‐HZ10 increased AhR expression levels by suppressing its CUL4B‐mediated ubiquitination degradation. Thus, lnc‐HZ10 and AhR formed a positive feedback loop. As a transcription suppressor, AhR suppressed BRCA1 transcription and down‐regulated its protein levels. Meanwhile, the first segment of lnc‐HZ10 (lnc‐HZ10‐S1) interacted with γ‐H2AX and impaired the recruitment of BRCA1 to DSB foci through γ‐H2AX. In normal trophoblast cells, AhR/lnc‐HZ10 feedback loop was lowly expressed. As a result, BRCA1 was highly expressed and was efficiently recruited to DSB foci to perform HR repair, giving normal pregnancy. Upon BPDE exposure, AhR was activated and promoted lnc‐HZ10 transcription. Thus, BPDE exposure triggered this AhR/lnc‐HZ10 feedback loop, which subsequently suppressed HR repair and possibly induced miscarriage. The molecular mechanisms were consistent in BPDE‐exposed human trophoblast cells and in RM villous tissues. In mouse system, due to lack of its corresponding lnc‐HZ10, it might be some other approaches to up‐regulate Ahr expression levels. Knockdown of murine Ahr could efficiently recover HR repair in placental tissues and alleviate miscarriage in BaP‐exposed mouse miscarriage model. Collectively, these results supported that BaP/BPDE suppressed HR repair, and then, further induced miscarriage.

### 3.3 Regulation of BRCA1

BRCA1 is a crucial protein for HR repair.^[^ ^26^ ^]^ It was found that AhR could bind with BRCA1 promoter and inhibit BRCA1 transcription in MCF7, UACC3199, and HCC38 BC cell lines.^[^ ^49^ ^]^ In tongue cell carcinoma (TSCC) tumors, cisplatin‐sensitivity‐associated lncRNA (CISAL) controlled mitochondrial fission and cisplatin sensitivity by inhibiting BRCA1 transcription in TSCC models.^[^ ^69^ ^]^ LncRNA AY343892 inhibited breast cancer development by positively regulating BRCA1‐mediated transcription of PTEN.^[^ ^70^ ^]^ In human trophoblast cells, woman villous tissues, and mouse placental tissues, we further confirmed that AhR bound with BRCA1 promoter region and suppressed its transcription. Thus, BRCA1 was down‐regulated by AhR/lnc‐HZ10 feedback loop, which was further triggered by BaP/BPDE exposure, providing a new approach to regulate the expression of BRCA1 in human cells.

### 3.4 BRCA1 Recruitment

Recruitment of BRCA1 to DSB foci by γ‐H2AX is a critical step for HR repair.^[^ ^31^, ^32^ ^]^ In this work, we found that BRCA1 could bind with γ‐H2AX in human trophoblast cells, villous tissues, and mouse placental tissues. Moreover, lnc‐HZ10, especially its first segment, directly interacted with γ‐H2AX and impaired its binding with BRCA1; thus, possibly reducing its recruitment to DSB foci and finally suppressing HR repair. The results revealed a novel epigenetic pathway to regulate BRCA1 recruitment in human cells. In addition to protein interactions of BRCA1 with γ‐H2AX, BRCA1 also involved in other functions in HR repair. BRCA1 interacted with BRCA1‐associated RING domain protein 1 (BARD1) to mediate the initial nucleolytic resection of DNA lesions and the recruitment and regulation of the recombinase RAD51.^[^ ^71^ ^]^ The BRCT domain of BRCA1 interacted with CtIP‐interacting protein (CtIP), which was involved in DNA end resection.^[^ ^72^, ^73^ ^]^ The coiled‐coil domain of BRCA1 interacted with BRCA2‐DSS1, which promoted the binding of BRCA1‐BARD1 complex to DNA lesion.^[^ ^74^, ^75^ ^]^ Therefore, whether these processes were also involved in the occurrence of miscarriage and were regulated by lnc‐HZ10 is also worthy of further exploration.

### 3.5 BPDE Exposure Activated AhR Expression

Increasing attention has been paid on environmental toxicants and human reproductive health. Although lots of studies have shown that exposure of environmental toxicants alter the expression profiles of mRNAs and non‐coding RNAs,^[^ ^76^ ^]^ the underlying mechanisms have been rarely explored. In this work, we found that exposure of BaP/BPDE (a typical aryl hydrocarbon) activated the expression of AhR (an aryl hydrocarbon receptor) in human trophoblast cells. AhR could act as a transcription factor to promote, or as a suppressor to suppress the transcription of its target genes. Herein, we identified that AhR promoted lnc‐HZ10 transcription but suppressed BRCA1 transcription in human trophoblast cells. Further, we hypothesized that, upon BaP/BPDE exposure, AhR might regulate the transcription of a vast number of RNAs (including non‐coding RNAs), and so, alter the expression profiles of mRNAs and non‐coding RNAs in BPDE‐exposed human trophoblast cells.

### 3.6 Miscarriage Intervention

It has been reported that lncRNAs could be used as targets for cancer therapy. For example, LINC00301 was highly expressed in non‐small cell lung cancer (NSCLC) tumor cells; and knockdown of LINC00301 by injecting its antisense locked nucleic acid (LNA) could efficiently reduce tumor size and volume in mouse model, providing a promising strategy for treatment against NSCLC.^[^ ^77^ ^]^ The recruitment of BRCA1 onto DSB foci is a crucial step for efficient HR repair.^[^ ^65^ ^]^ Moreover, knockdown of oncogenic lncRNA nuclear paraspeckle assembly transcript 1 (NEAT1) by novel LNA‐gapmeR antisense oligonucleotide inhibited multiple myeloma cell proliferation and also triggered cell apoptosis.^[^ ^78^ ^]^ As for miscarriage; although some studies have shown extensive regulatory mechanisms of miscarriage,^[^ ^79^, ^80^ ^]^ miscarriage intervention strategy has been rarely reported. In this study, we used AS‐Ahr to intervene miscarriage in BaP‐exposed mouse miscarriage model and found that this approach could efficiently alleviate miscarriage in this model. These results not only further validated the molecular mechanism underlying miscarriage but also provided an approach for prevention and treatment against unexplained recurrent miscarriage.

### 3.7 Limitation of This Work

In this study, we focused on the regulatory roles of lnc‐HZ10 in the HR repair of DSB. Although we found that lnc‐HZ10 had little effects on NHEJ repair, the potential effects of lnc‐HZ10 on other DNA repair, such as base excision repair or mismatch repair, should be further considered. Further, there might be NHEJ repair of DSB in villous tissues, which might be regulated by other lncRNAs and might also be associated with miscarriage. These hypotheses should be further investigated. γ‐H2AX accumulation was dependent on both the generation of DSB and the reduction in HR repair, both of which might be regulated by BaP/BPDE exposure and should be further explored. Moreover, more experiments might be required to further confirm the consistence of the results in vivo and in vitro. Lnc‐HZ10 homology was not observed in mouse. Murine lncRNAs that might regulate HR repair in mouse might be further explored. Knockdown of Ahr alleviated miscarriage in mouse model, which still required more experiments before application in the human system. In addition, BRCA1 could also function as ubiquitin E3 ligase, which took on an important role in HR repair.^[^ ^81^, ^82^, ^83^ ^]^ After recruitment of BRCA1 by γ‐H2AX, its ubiquitination functions in HR repair should be further explored. Moreover, BRCA1 forms a heterodimer with BARD1.^[^ ^83^ ^]^ The regulatory roles of lnc‐HZ10 in the expression levels of BARD1 and its protein interactions with BRCA1 or γ‐H2AX should also be investigated.

In summary, we described a novel lnc‐HZ10 that suppressed HR repair of DSB in human trophoblast cells. Lnc‐HZ10 and AhR formed a positive feedback loop. AhR suppressed BRCA1 transcription; and lnc‐HZ10 interacted with γ‐H2AX to impair its interactions with BRCA1. In normal trophoblast cells, the AhR/lnc‐HZ10 loop was lowly expressed to activate HR repair, giving normal pregnancy. However, BPDE exposure might trigger this loop in cells and suppress HR repair, possibly inducing miscarriage. Assays in BPDE‐exposed trophoblast cells and RM tissues gave the similar regulatory mechanisms. Knockdown of murine Ahr could efficiently recover HR repair in placental tissues and alleviate miscarriage in the BaP‐exposed mouse miscarriage model. Based on these observations, we suggested that the AhR/lnc‐HZ10/BRCA1 axis might be considered as a promising target for treatment against unexplained miscarriage.

## 4 Experimental Section

### Materials

BPDE (purity 99.9%), purchased from MRIGlobal Chemical Co. (Kansas, MO, USA), was dissolved in anhydrous DMSO (Sigma–Aldrich, St Louis, MO, USA). BaP (purity 99.9%, Sigma–Aldrich) was diluted to 50 µg mL^−1^ in corn oil (Sigma–Aldrich). Cyclohexide (CHX, Cat. No. M4879) and MG132 (Cat. No. M1902) were purchased from AbMole Bioscience (Houston, Texas, USA).

### Cell Culture

Swan 71 cells, immortalized by human telomerase, were constructed by Gil Mor lab at Yale University.^[^ ^84^ ^]^ HTR‐8/SVneo cells were commercially available (CRL‐3271, ATCC, USA). Swan 71 cells and HTR‐8/SVneo cells were, respectively, cultured in DMEM medium (Gibco, Invitrogen, Carlsbad, CA, USA) and RPMI 1640 medium (Gibco, Invitrogen, Carlsbad, CA, USA) supplemented with 10% FBS (fetal bovine serum) at 37 °C with 5% v/v CO_2_. Swan 71 cells were also synchronized in the G2 phase using double‐thymidine block and release protocols.^[^ ^85^ ^]^ Briefly, trophoblast cells were incubated in medium containing 2 mm thymidine (Sigma–Aldrich) for 18 h; then, in fresh medium for 9 h; and then, incubated in medium containing 2 mm thymidine for 18 h to synchronize cells in the S phase. Subsequently, the cells were incubated in fresh medium for 5 h to synchronize cells in the G2 phase, which were further verified by flow cytometry analysis.

### Tissue Collection and Statement

Human villous tissues were collected from 20 women who had elective abortion as healthy control (HC group) and 20 patients with unexplained RM group with two or more consecutive unexplained spontaneous miscarriages at 6–10 weeks of gestation, as the methods described previously.^[^ ^7^, ^8^, ^12^, ^13^, ^14^, ^15^, ^38^ ^]^ They were all aged 25–30 years old. All of the HC group had previous successful pregnancies. Any woman in both groups with one of the following characteristics was excluded: abnormal uterus and cervix, abnormal karyotype, autoimmune deficiency, abnormal hormone secretion, metabolic diseases, viral infectious diseases, endocrine, and so on, as described previously.^[^ ^7^, ^8^, ^12^, ^13^, ^14^, ^15^, ^38^ ^]^ RNAs and proteins were extracted from these tissues and analyzed by RT‐qPCR and Western blotting, respectively. The experiment protocols had been approved by the Eighth Affiliated Hospital, Sun Yat‐sen University (Approval no. 2022‐017‐01). Written informed consent had been signed before the study.

### Cell Transfection

Swan 71 cells or HTR‐8/SVneo cells (1 × 10^6^ cells per well) were seeded into six‐well plates for 24 h, and then, transfected with negative control, overexpression plasmid (Sangon Biotech, China), or siRNA (Invitrogen, USA) in Lipofectamine 3000 (L300015, Invitrogen) for 6 h, and then, incubated for 24 h in a new media culture. Subsequently, the cells were harvested for analysis. The transfection efficiencies were validated by qPCR. The sequences of siRNAs are listed in Table S1, Supporting Information, and genes in plasmids are listed in Table S2, Supporting Information.

### 4.1 and 3′ Rapid Amplification of cDNA Ends (RACE)

RACE assays were conducted using the SMARTer RACE 5′/3′ Kit (Takara Bio, Tokyo, Japan), as described previously.^[^ ^7^, ^8^, ^10^ ^]^ After treatment of cell lysate with DNase to degrade DNA, total RNAs were extracted using the RNeasy Micro Kit (Bio‐Rad, Hercules, CA, USA). Total RNAs were reverse transcripted to cDNAs. Then, 5′‐ and 3′‐RACE PCRs were performed using the universal and gene specific primers (sequences in Table S3, Supporting Information). Subsequently, PCR products were cloned into linearized pRACE vector for DNA sequencing.

### FISH

Swan 71 cells were cultured on sterile glass slides and incubated with probe overnight. Cy5‐conjugated ssDNA probe (5′‐Cy5‐TTCCTGTCGTCCGTCCCTTGCGGTG‐3′) was customized from GenePharma (Shanghai, China) and was used to target lnc‐HZ10. Images (600×) were captured on a confocal laser scanning microscope (N‐STORM+A1R, Nikon, Japan). The relative expression levels of lnc‐HZ10 in the nucleus and cytoplasm were calculated using Image J.

### Immunofluorescence Assay

Trophoblast cells were seeded on coverslips for 24 h, fixed with 4% paraformaldehyde (P1110, Solarbio) for 15 min; and then, permeabilized with 0.5% Triton X‐100 (T8200, Solarbio) for 20 min on ice. Cells on coverslips were incubated with anti‐BRCA1 (1:50, MS110, Invitrogen), anti‐RAD51 (1:50, MA1‐23271, Invitrogen), anti‐AhR (1:50, MA1‐513, Invitrogen), or anti‐γ‐H2AX (1:50, ab20669, Abcam) antibody at 4 °C overnight; and subsequently, incubated with Alexa Fluor 594 (B40942, Invitrogen)‐conjugated or Alexa Fluor 488 (B40943, Invitrogen)‐conjugated secondary antibodies at 37 °C for 20 min. The nuclei were stained with DAPI. The coverslips were visualized on a confocal fluorescence microscope (N‐STORM+A1R, Nikon, Japan).

### Western Blotting

Total proteins were extracted and protein concentrations were determined as described previously.^[^ ^7^, ^8^, ^12^, ^13^, ^14^, ^15^, ^38^ ^]^ Primary antibodies included anti‐BRCA1 (1:1000, MS110, Invitrogen), anti‐53BP1 (1:5000, PA1‐16565, Invitrogen), anti‐Ku70 (1:1000, PA5‐27538, Invitrogen), anti‐Ku80 (1:1000, PA5‐17454, Invitrogen), anti‐DNA‐PKcs (1:500, MA5‐15813, Invitrogen), anti‐TopBP1 (1:1000, PA5‐96424, Invitrogen), anti‐PARP1 (1:1000, ab191217, Abcam), anti‐PARP2 (1:1000, ab271080, Abcam), anti‐AhR (1:1000, MA1‐513, Invitrogen), anti‐CUL4B (1:1000, PA5‐51084, Invitrogen), anti‐γ‐H2AX (1:5000, ab20669, Abcam), anti‐β‐Tubulin (1:5000, ab250104, Abcam), and anti‐H3 (1:5000, ab1791, Abcam). Secondary antibodies included HRP‐conjugated anti‐rabbit IgG (ab150077, Abcam) and HRP‐conjugated anti‐mouse IgG (ab205719, Abcam). Image J was used for quantification of the intensity of Western blot bands. β‐Tubulin protein band was used as loading control. For better comparison of the protein bands in control and experiment groups, different amount of cell lysates was loaded in different experiments.

### Reverse Transcription and Quantitative Real‐Time PCR (RT‐qPCR)

The first strand cDNA synthesis kit (Takara, Kyoto, Japan) was used for synthesis of cDNAs. Real‐time quantitative PCR and data analysis were performed on a CFX96 instrument (Bio‐Rad) with SYBR Green reagent (Bio‐Rad). Relative gene expression was analyzed by the comparative 2^−ΔΔCt^ method. The primer sequences are shown in Table S4, Supporting Information.

### Immunoprecipitation (IP) and Co‐IP Assays

Swan 71 or HTR‐8/SVneo cells were lyzed in IP lysis buffer containing protease inhibitors for 15 min on ice and centrifugated at 14000 × *g* for 15 min at 4 °C. The equal volume of cell lysates was incubated with the indicated antibody (including anti‐IgG [1:50, ab205719, Abcam], anti‐BRCA1 [1:50, MS110, Invitrogen], or anti‐γ‐H2AX [1:50, ab20669, Abcam]) at 4 °C overnight. Meanwhile, an aliquot of cell lysates was used to indicate the original protein levels. In order to reduce the influence of proteins in cell lysates on the protein interactions between γ‐H2AX and BRCA1, a limited but identical amount of γ‐H2AX or BRCA1 antibody was used in Co‐IP assays. Then, the resulting mixture was incubated with protein A/G immunoprecipitation magnetic beads (B23201, Bimake) at 4 °C for 1 h. The beads were washed thrice with lysis buffer. The immunoprecipiated proteins were separated from beads and analyzed by Western blotting.

### Ubiquitination Assay

Trophoblast cells were treated with MG132 (M1902, AbMole Bioscience, USA) for 6 h, and then lyzed, in lysis buffer containing protease inhibitors. Cell lysates were incubated with 30 µL Protein A/G immunoprecipitation magnetic beads (B23201, Bimake), coupling with anti‐AhR antibody (1:250, PA5‐122264, Invitrogen) or anti‐IgG antibody at 4 °C overnight. Subsequently, the ubiquitinated AhR (AhR‐Ub) was detected by Western blotting.

### Homologous Recombination (HR) or Non‐Homologous End Joining (NHEJ) Repair Assays

Homologous recombination (HR) or non‐homologous end joining (NHEJ) repair assays were conducted as described previously.^[^ ^86^ ^]^ Briefly, Swan 71 or HTR‐8/SVneo cells were transfected with siRNA‐HZ10, siRNA‐BRCA1, siRNA‐AhR, siRNA‐NC, pcDNA3.1‐HZ10, pcDNA3.1‐BRCA1, pcDNA3.1‐AhR, or pcDNA3.1 empty vector, together with DR‐GFP plasmid or EJ5‐GFP plasmid, and I‐SecI plasmid, in Lipofectamine 3000 for 8 h. After incubation for another 72 h, DR‐GFP‐positive cells (cells with HR) or EJ5‐GFP‐positive cells (cells with NHEJ) were determined by flow cytometry (FACSCanto, BD Biosciences) with FlowJo Software.

### Comet Assays

The comet assays were performed using Comet Assay Kit (ab238544, Abcam) according to the manufacturer's protocols. Briefly, Swan 71 or HTR‐8/SVneo cells were transfected with siRNA‐HZ10, siRNA‐NC, pcDNA3.1‐HZ10, or empty vector and harvested in PBS. Then, these cells were embedded and lyzed in 0.5% low‐melting‐point agarose under alkaline conditions (pH > 13). Embedded DNA was analyzed by agarose electrophoresis. Afterward, DNA was stained with Green DNA Dye. Images were captured using a upright fluorescence microscope (ECLIPSE Ci, Nikon, Japan).

### Protein Stability Assay

Trophoblast cells were treated with 70 µg mL^−1^ cycloheximide (M4879, AbMole) to block protein translation. After incubation for 0, 2, 4, 6, or 8 h, the remaining AhR protein in trophoblast cells was analyzed by Western blotting.

### RNA Stability Assay

Trophoblast cells were treated with 5 µg mL^−1^ actinomycin D (Act D, M4881, AbMole) to block RNA transcription. After incubation for 0, 2, 4, 6, 8, or 10 h, the remaining RNA in trophoblast cells was analyzed by RT‐qPCR.

### Chromatin Immunoprecipitation (ChIP) Assay

ChIP assays were conducted with the EZ‐Magna ChIP Chromatin Immunoprecipitation Kit (Cat. No. 17–408, Millipore). Cells were cross‐linked with 1% formaldehyde at 25 °C for 20 min and lyzed in SDS buffer. Chromatin was pelleted and re‐suspended in immunoprecipitation buffer for sonication on ice to mildly shear dsDNA into 200–1000 bp fragments. Then, the mixture was incubated with Protein A/G immunoprecipitation magnetic beads, coupling with AhR or IgG antibody at 4 °C overnight. After separation of these beads, DNA bound onto beads was extracted and analyzed by qPCR. The primer sequences are listed in Table S5, Supporting Information.

### RNA Immunoprecipitation (RIP)

RNA Immunoprecipitation Kit (17‐704, Millipore, Burlington, MA, USA) was used for RIP assays. Briefly, trophoblast cell lysates were incubated with magnetic beads conjugated with antibodies of IgG (1:50, anti‐IgG), γ‐H2AX (1:50, anti‐γ‐H2AX), or BRCA1 (1:50, anti‐BRCA1) overnight at 4 °C. RNAs enriched by proteins on beads were extracted and analyzed by RT‐qPCR. For RIP‐Rnase assays, trophoblast cell lysates were treated with 1 U µL^−1^ RNase T1 to degrade the unbound regions of lnc‐HZ10 before RIP assays. Then, the fragments of lnc‐HZ10 bound with protein were eluted and analyzed as described above. The primer sequences are shown in Table S4, Supporting Information.

### Biotinylated RNA Pulldown Assay

Biotinylated RNA pulldown assays were performed as described previously.^[^ ^7^ ^]^ Briefly, oligonucleotides of lnc‐HZ10 or its various segments were designed, synthesized, and in vitro transcribed from pGEM‐T‐lnc‐HZ10 or pGEM‐T‐lnc‐HZ10‐S1‐5 (Addgene) (sequences in Table S6, Supporting Information). All the transcripts were biotin‐labeled by T7 RNA polymerase using Biotin RNA Labeling Mix (Roche, Basel, Switzerland). After treatment with DNase I, the RNAs were purified with RNeasy Mini Kit (Qiagen, Valencia, CA, USA). The transcripts were then incubated with trophoblast cell lysates at 4 °C overnight. Finally, the protein‐RNA complexes were separated from streptavidin magnetic beads, and the proteins were analyzed by Western blotting with anti‐BRCA1 (1:1000, MS110, Invitrogen) or anti‐γ‐H2AX (1:1000, ab20669, Abcam) antibody.

### Nuclear/Cytoplasmic Fractionation Assay

Nuclear/cytoplasmic fractionation assays were conducted with NE‐PER Nuclear and Cytoplasmic Extraction Reagents (78835, Millipore, Burlington, MA, USA). Swan 71 or HTR‐8/SVneo cells were lyzed with a homogenizer in cold cytoplasmic lysis buffer on ice for 10 min. The supernatant was collected by centrifugation at 15 000 × *g* for 15 min at 4 °C as cytoplasmic proteins. The precipitate was re‐suspended in nuclei lysis buffer and vortexed for 30 min at 4 °C. Then, the supernatant was collected by centrifugation at 15 000 × *g* for 15 min at 4 °C as nuclear proteins. The cytoplasmic and nuclear proteins were analyzed by Western blotting, with H3 as nuclear marker and β‐Tubulin as cytoplasmic marker.

### Dual‐Luciferase Reporter Assay

Wild‐type (wt) or mutant (mt) promoter sequence of BRCA1 or lnc‐HZ10 was fused into the luciferase pmirGLO‐basic reporter vector (Madison, Promega, USA). Swan 71 cells were co‐transfected with 300 ng pmirGLO‐wt/mt‐BRCA1 or pmirGLO‐wt/mt‐HZ10 and 200 nm AhR plasmid or its vector in Lipofectamine 3000 (Invitrogen). Luciferase activity was measured using Dual‐Luciferase Reporter assays (E1910, Promega, USA). Briefly, trophoblast cells were incubated for 24 h and lyzed in passive lysis buffer on ice for 20 min. Cell lysates were then mixed with luciferase assay reagent. The firefly luciferase activity was measured on a fluorescence analyzer (PE envision, PE, China). Subsequently, cell lysates were incubated with stop reagent and the renilla luciferases activity was measured as background value.

### Mouse Model

A pregnant mouse model with BaP‐induced miscarriage was constructed as described previously.^[^ ^7^, ^12^, ^13^, ^14^, ^15^ ^]^ Briefly, pregnant C57BL/6 mice (Charles River Company, Beijing, China) were randomly divided into three groups (each *n* = 6) and daily given 0, 0.05, or 0.2 mg kg^−1^ BaP by oral gavage from D1 to D13. To construct a miscarriage intervention model, pregnant C57BL/6 mice were randomly divided into four groups (each, *n* = 6) : 1) corn oil group, 2) BaP group, 3) BaP + AS‐NC group, and 4) BaP + AS‐Ahr group. Four groups were daily given 0.2 mg kg^−1^ BaP or corn oil from D1 to D13 by oral gavage. In addition, the mice were also intraperitoneally injected with 20 mg kg^−1^ AS‐Ahr (antisense oligonucleotides targeting murine Ahr mRNA) or AS‐NC (negative control) once per 3 days from D1 to D13. Sequences of AS‐Ahr and AS‐NC are shown in Table S7, Supporting Information. All the mice were euthanized by injection with nembutal (100 mg kg^−1^) for collection of uterus on D14. Miscarriage rate in each mouse was calculated as the number of embryo resorptions divided by the number of total embryos. A random placenta was collected from every mouse in each group for RNA and protein extraction. The animal protocols had been authorized by the Medical Ethics Committee of West China Medical Center, Sichuan University (Approval no. K2019004‐2).

### Statistical Analysis

Every experiment was performed independently thrice with similar results, and the data were expressed as mean ± SD (standard deviation, *n* = 3). Statistical analysis was performed using SPSS v24.0 software or Prism GraphPad 8.0. The significance of differences between two groups (*n* = 3) was determined using the Kruskal–Wallis test. The significance of differences between three or more groups (*n* = 3) was determined using the Kruskal–Wallis test followed by Wilcoxon pairwise comparisons. The significance of differences between two groups (parametric data) was determined using the unpaired Student's *t*‐test. The significance of differences between three or more groups (parametric data) was analyzed by ANOVA tests followed by Tukey's multiple comparisons test. The correlation was analyzed by Pearson analysis. *, *p* < 0.05; **, *p* < 0.01; and *p* < 0.05 were considered as significant.

## Conflict of Interest

The authors declare no conflict of interest.

## Author Contributions

W.C.and H.Z. designed the study. W.C. performed most of the experiments. W.C. and H.Z. wrote the draft manuscript. W.C., C.M., Y.Z., and Y.Y were responsible for animal experiments. W.H. and Z.X. contributed to the tissue preparation. J.Z., R.W., M.W., S.W., and X.W. conducted the remaining experiments.

## Supporting information

Supporting Information

## Data Availability

The data that support the findings of this study are available from the corresponding author upon reasonable request.
