# Supplementary material for: Defective Homologous Recombination Repair By Up‐Regulating Lnc‐HZ10/Ahr Loop in Human Trophoblast Cells Induced Miscarriage
Source: Adv Sci (Weinh). 2024 Jan 29;11(13):2207435. doi: 10.1002/advs.202207435 (PMC10987163; doi:10.1002/advs.202207435)
Supplement: Supplementary file 1 — Supporting Information [file ADVS-11-2207435-s001.pdf]

ADVANCED SCIENCE
Open Access

Supporting Information

for *Adv. Sci.*, DOI 10.1002/advs.202207435

Defective Homologous Recombination Repair By Up-Regulating Lnc-HZ10/Ahr Loop in Human Trophoblast Cells Induced Miscarriage

*Weina Chen, Chenyang Mi, Ying Zhang, Yang Yang, Wenxin Huang, Zhongyan Xu, Jingsong Zhao, Rong Wang, Manli Wang, Shukun Wan, Xiaoqing Wang and Huidong Zhang**

Supplementary Materials

Defective Homologous Recombination Repair by Up-regulating Lnc-HZ10/AhR Loop in Human Trophoblast Cells Induced Miscarriage

Weina Chen, Chenyang Mi, Ying Zhang, Yang Yang, Wenxin Huang, Zhongyan Xu, Jingsong Zhao, Rong Wang, Manli Wang, Shukun Wan, Xiaoqing Wang and Huidong Zhang*

Table S1. Sequences of siRNAs for cell transfection.

Name	Sense (5'-3')	Antisense (5'-3')
si1-HZ10	CCAACAGAGGUGUGGGAAATT	UUUCCCACACCUCUGUUGGTT
si2-HZ10	GGGAAGAGCUCCUGUACGATT	UCGUACAGGAGCUCUUCCCTT
si1-BRCA1	GAGAAUCCUAGAGAUACUGAATT	UUCAGUAUCUCUAGGAUUCUCTT
si2-BRCA1	GCCUACAAGAAAGUACGAGAUTT	AUCUCGUACUUUCUUGUAGGCTT
si1-AhR	UUGUACUACACAAAAGUACUGTT	CAGTACUUUUGUGUAGUCAATT
si2-AhR	UAGTACUUUUGUGUAGUCAATT	UUGUACUACACAAAAGUACUATT
si1-CUL4B	CCACCCAGAAGTCATTAATTT	AUUAAUGUCUUCUGGGUGGTT
Si2-CUL4B	GGCAGCACUAUUGUAAUUATT	UAAUUACAAUAGUGCUGCCT
siRNA NC	UUCUCCGAACGUGUCACGUTT	ACGUGACACGUUCGGAGAATT

Table S2. DNA sequences used for construction of pcDNA3.1 overexpression plasmids.

Gene	Sequence region (5'-3')

Lnc-HZ10	Full length of NCBI No. OK315569, chr 16: 3,156,683 - 3,158,734, 1~2052 nt
Lnc-HZ10-S1	TCACAGGAAG⋯CAGCCCTGGG, chr 16: 3,156,683 - 3,157,130, 1~447 nt
Lnc-HZ10-ΔS1	CCCTGGGACC⋯ATCCAGCCTG, chr 16: 3,157,130 - 3,158,734, 447~2052 nt
BRCA1	NM_001407571.1 CDS region
AhR	NM_001621.5 CDS region
CUL4B	NM_001079872.2 CDS region

Table S3. Primers used for 5′- and 3′-RACE assays.

Primer	Sequence region (5'-3')
5'-RACE of lnc-HZ10	CTCGGTGCTAAAACCTGCCT
3'-RACE of lnc-HZ10	AGTGTCCTTCGACCATCCTCC

Table S4. Primer sequences used for RT-qPCR analysis.

Species	Gene	Forward (5'-3')	Reverse (5'-3')
Human	Lnc-HZ10	GGAAACGCGCTCAGAAGTTG	ATCTAACTCGAGGTGCTCGC
	AhR	AGGGCGAAAGAGAAAAGCGA	TGGACAGGTCAAATACCGCC
	BRCA1	TCGGTCCCTCAGAACACGA	AAACGCGGAGAAACGGGAC
	H2AX	TTCACCGGTCTACCTCGCTA	CGGGCCCTCTTAGTACTCCT
	GAPDH	TGTGTCCGTCGTGGATCTGA	GCAGCTGTGACACACAGTA
	lnc-HZ10-1 (1-447 nt)	TCACAGGAAGCTGGTAGGAG	AGAAGGAGCTCGGCTGAGGA
	lnc-HZ10-2 (440-842 nt)	GGAGCTGGGGTCAGAGTTCA	TGCCGTCCCTTGCGGTG
	lnc-HZ10-3 (820-1236 nt）	CAGGAAACACACACCCTCG	AGCTCTCCCGAACTTCCCTAT
	lnc-HZ10-4	GGGAATTGTTGGTCAGGCAA	ACAAGACAAAGGGGATCCTGA

	(1236-1721 nt)		
	lnc-HZ10-5 (1718-2052 nt)	TGTGCCAGTGCATCCAGCCTG	CACCATGTTAGCCAGGATGG
Mouse	Brca1	CGAATCTGAGTCCCCTAAAGAGC	AAGCAACTTGACCTTGGGGTA
	Ahr	GCCCTTCCCGCAAGATGTTAT	TCAGCAGGGGTGGACTTTAAT
	H2ax	CGGTGGGCTTGAAGGTTAGT	ACTGGTATGAGGCCAGCAAC
	Gapdh	AGGTCGGTGTGAACGGATTTG	GGGGTCGTTGATGGCAACA
	Sequence No. 1	ATTGTTGGTCAGGCAAACGC	TCCTGAAGCCAGTTGGTTGG
	Sequence No. 2	CCACCATGGCTCTAAGACAAGT	TCCCCACAGACCTCTTGGAAT

Table S5. Sequences of the primers used in various ChIP PCR assays.

Name	Forward primer (5'-3')	Reverse primer (5'-3')
BRCA1	CTCCCATCCTCTGATTGTACCTTGAT	CAGGAAGTCTCAGCGAGCTCAC
Lnc-HZ10	GGAATCCGCGTGAGTCTTGA	ACTCCTCGAAGTGCCTCAGA
GAPDH	TACTAGCGGTTTTACGGGCG	TCGAACAGGAGGAGCAGAGAGCGA

Table S6. DNA sequences used for construction of pGEM-T plasmids

Gene	Sequence region (5'-3')
Lnc-HZ10	Full length of NCBI No. OK315569, chr 16: 3,156,683 - 3,158,734, 1~2052 nt
Lnc-HZ10-S1	TCACAGGAAG···CAGCCCTGGG, chr 16: 3,156,683 - 3,157,129, 1~447 nt
Lnc-HZ10-S2	CCCTGGGACC···CACACCCTCG, chr 16: 3,157,123 - 3,157,524, 440~842 nt
Lnc-HZ10-S3	AGACAGGAAA···AGAGCTCCAA, chr 16: 3,157,502- 3,157,918, 820~1236 nt
Lnc-HZ10-S4	CAGAGGTGTG···CCGAGACTGT, chr 16: 3,157,893 - 3,158,403, 1210~1721 nt
Lnc-HZ10-S5	TGTGCCAGTG···ATCCAGCCTG, chr 16: 3,157,401 - 3,158,734, 1718~2052 nt

Table S7. Sequences of antisense oligonucleotides used in mouse miscarriage intervention.

Gene	Sequence (5'-3')
AS-Ahr	lT*lA*lT*(dT)*(dA)*(dA)*(dT)*(dA)*(dA)*(dC)*(dA)*(dT)*(dC)*(dT)*(dT)*(dG)*lC*lG*lG
AS-NC	lT*lT*lA*C*G*C*A*A*T*C*T*T*C*T*C*G*lA*lG*lG

*Phosphorothioated oligonucleotides

Table S8. The sequences of two fragments in mouse genome that have similar sequences as human lnc-HZ10.

Fragment	Sequence region of human lnc-HZ10 (5'-3')	Identity	Strand	Sequence region in mouse genome (5'-3')	Length, nt
**No. 1**	chr 16: 3,157,942 - 3,158,061	73.3%	+	chr 16: 47,250,547 - 47,250,666	120
**No. 2**	chr 16: 3,157,939 - 3,158,013	98.7%	+	chr 17: 23,547,358 - 23,547,432	75

Figure legends

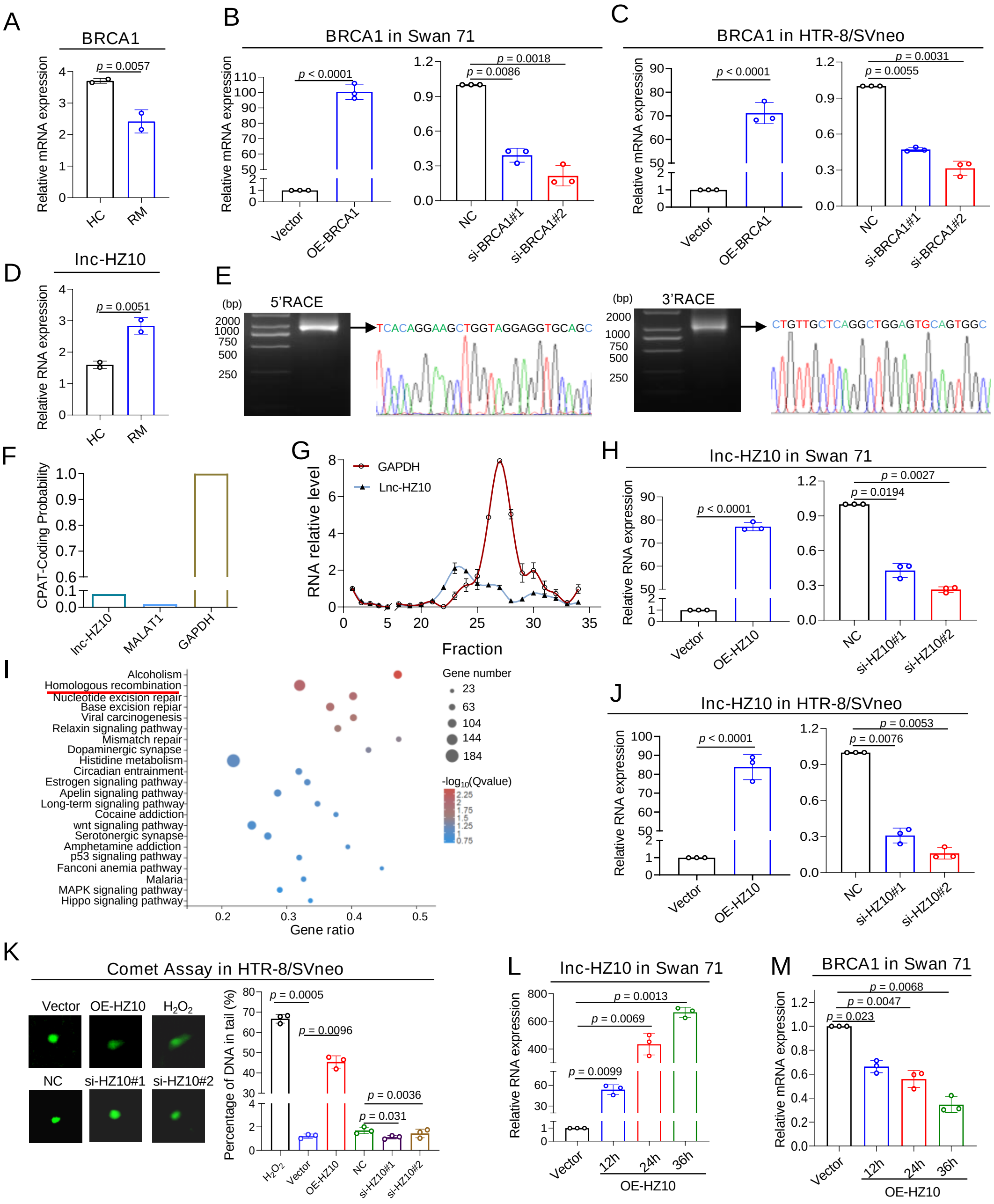
A
BRCA1
Relative mRNA expression
p = 0.0057
HC
RM
B
BRCA1 in Swan 71
p < 0.0001
Vector
OE-BRCA1
p = 0.0018
p = 0.0086
NC
si-BRCA1#1
si-BRCA1#2
C
BRCA1 in HTR-8/SVneo
p < 0.0001
p = 0.0031
p = 0.0055
D
lnc-HZ10
Relative RNA expression
p = 0.0051
E
(bp)
5'RACE
3'RACE
TCACAGGAAGCTGGTAGGAGGTGCAGC
CTGTTGCTCAGGCTGGAGTGCAGTGGC
F
CPAT-Coding Probability
lnc-HZ10
MALAT1
GAPDH
G
GAPDH
Lnc-HZ10
RNA relative level
Fraction
H
lnc-HZ10 in Swan 71
p < 0.0001
p = 0.0027
p = 0.0194
Vector
OE-HZ10
si-HZ10#1
si-HZ10#2
I
Alcoholism
Homologous recombination
Nucleotide excision repair
Base excision repair
Viral carcinogenesis
Relaxin signaling pathway
Mismatch repair
Dopaminergic synapse
Histidine metabolism
Circadian entrainment
Estrogen signaling pathway
Apelin signaling pathway
Long-term signaling pathway
Cocaine addiction
wnt signaling pathway
Serotonergic synapse
Amphetamine addiction
p53 signaling pathway
Fanconi anemia pathway
Malaria
MAPK signaling pathway
Hippo signaling pathway
Gene ratio
Gene number
-log10(Qvalue)
J
lnc-HZ10 in HTR-8/SVneo
p < 0.0001
p = 0.0053
p = 0.0076
K
Comet Assay in HTR-8/SVneo
Vector
OE-HZ10
H2O2
NC
si-HZ10#1
si-HZ10#2
Percentage of DNA in tail (%)
p = 0.0005
p = 0.0096
p = 0.0036
p = 0.031
L
lnc-HZ10 in Swan 71
p = 0.0013
p = 0.0069
p = 0.0099
12h
24h
36h
OE-HZ10
M
BRCA1 in Swan 71
p = 0.0068
p = 0.0047
p = 0.023

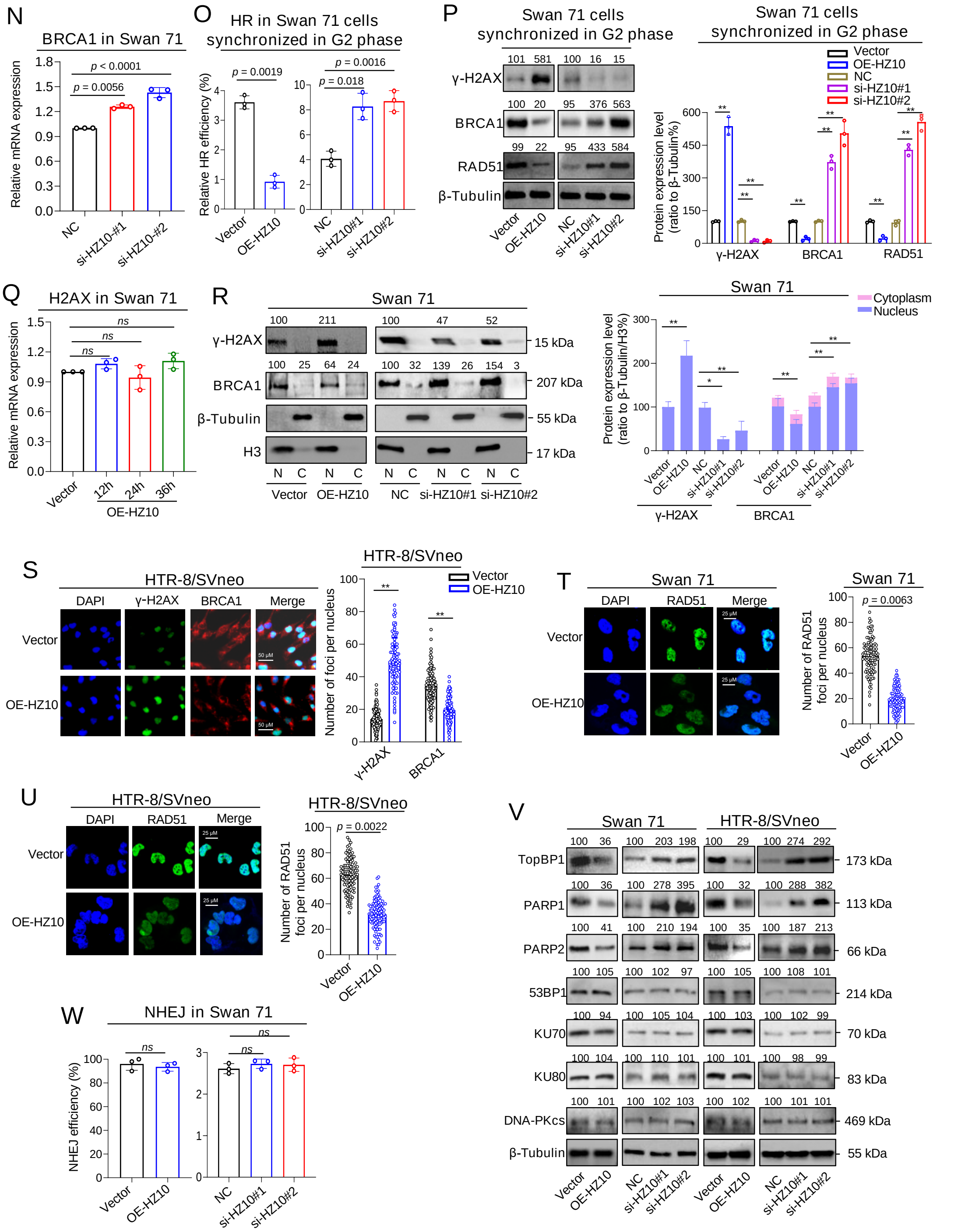

Figure. S1. Lnc-HZ10 was up-regulated in RM villous tissues and suppressed HR repair by down-regulating BRCA1 in human trophoblast cells.

(A) BRCA1 mRNA levels in transcriptome sequencing data of HC and RM villous tissues (each n = 2). **(B-C)** RT-qPCR analysis of BRCA1 mRNA levels in Swan 71 **(B)** or HTR-8/SVneo **(C)** cells with overexpression or knockdown of BRCA1. **(D)**

Lnc-HZ10 levels in transcriptome sequencing data of HC and RM villous tissues (each n = 2). **(E)** Rapid amplification of cDNA ends (RACE) assays showed that lnc-HZ10 was a transcript with a full length of 2,052 nucleotides (nt). **(F)** The protein-coding potential of lnc-HZ10 was predicted by Coding Potential Assessment Tool, with lncRNA MALAT1[1] as negative control and GAPDH mRNA as positive control. **(G)** Polyribosome binding assays showed that the binding of lnc-HZ10 with ribosome was very weak, with GAPDH mRNA as positive control. **(H)** RT-qPCR analysis of lnc-HZ10 in Swan 71 cells with overexpression or knockdown of lnc-HZ10. **(I)** KEGG pathway enrichment of the differentially expressed mRNAs in lnc-HZ10-overexpressed Swan 71 cells vs control cells. **(J)** RT-qPCR analysis of lnc-HZ10 levels in HTR-8/SVneo cells with overexpression or knockdown of lnc-HZ10. **(K)** Comet assay analysis and the relative quantification of DSB levels in HTR-8/SVneo cells with overexpression or knockdown of lnc-HZ10, with H_2O_2 as positive control. **(L-M)** RT-qPCR analysis of the levels of lnc-HZ10 (L) and BRCA1 mRNA (M) in Swan 71 with overexpression of lnc-HZ10 for 12, 24, or 36 hours. **(N)** RT-qPCR analysis of BRCA1 mRNA levels in HTR-8/SVneo with knockdown of lnc-HZ10. **(O)** Flow cytometry analysis of the relative HR efficiency in Swan 71 cells synchronized in G2 phase with overexpression or knockdown of lnc-HZ10. **(P)** Western blot analysis and the relative quantification of BRCA1, RAD51, and γ-H2AX protein levels in Swan 71 cells synchronized in G2 phase with overexpression or knockdown of lnc-HZ10, with β-Tubulin as loading control. **(Q)** RT-qPCR analysis of H2AX mRNA levels in Swan 71 cells with overexpression of

lnc-HZ10 for 12, 24, or 36 hours. **(R)** Nuclear/cytoplasmic fractionation assay analysis and the relative quantification of γ-H2AX and BRCA1 protein levels in nucleus and cytoplasm of Swan 71 cells, with β-Tubulin as cytoplasm marker and H3 as nucleus marker. **(S)** Immunofluorescence image analysis and the relative quantification of BRCA1 and γ-H2AX protein foci in HTR-8/SVneo cells with overexpression of lnc-HZ10. Scale bar = 50 μm. **(T-U)** Immunofluorescence image analysis and the relative quantification of RAD51 protein foci in Swan 71 (T) and HTR-8/SVneo (U) cells with overexpression of lnc-HZ10. Scale bar = 25 μm. **(V)** The protein levels of TopBP1, PARP1, PARP2, Ku70, Ku80, 53BP1, and DNA-PKcs in Swan 71 and HTR-8/SVneo cells with overexpression or knockdown of lnc-HZ10. **(W)** Flow cytometry analysis of relative NHEJ efficiency in Swan 71 cells with overexpression or knockdown of lnc-HZ10.

Representative data in (K left, P left, R-U left, V) represent three independent experiments. Data in (A-D, H, J, K right, L-O, P right, Q, R-U right, W) show mean ± SD of three independent experiments. Unpaired Student's *t*-test for (S-U right); Kruskal-Wallis test for (A, B-C left, D, H left, J left, O left, W left); Kruskal-Wallis test followed by Wilcoxon pairwise comparisons for (B-C right, H right, J-K right, L-N, O-Pright, Q, R right, W right). *, $p < 0.05$; **, $p < 0.01$; $p < 0.05$ was considered as significant.

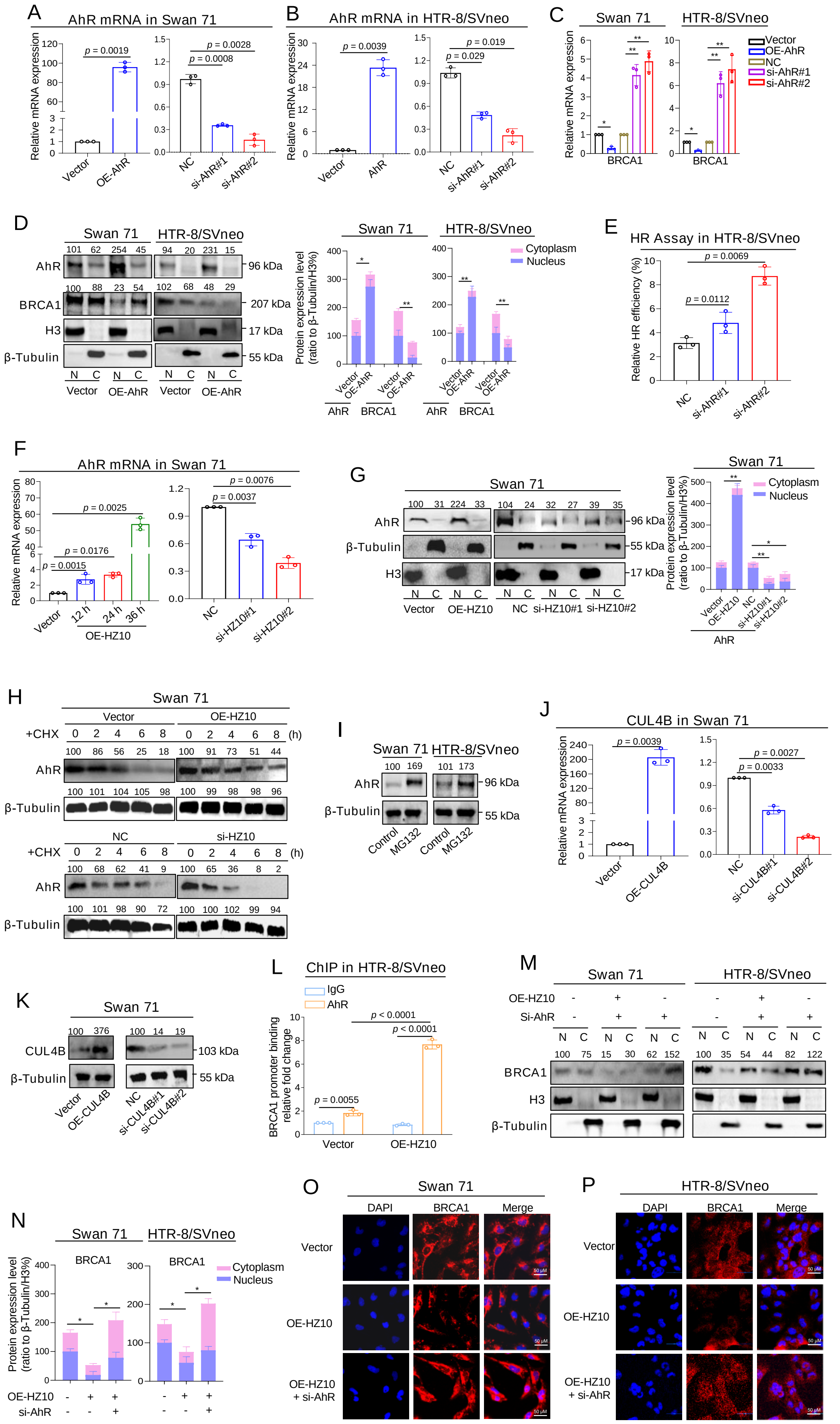
A
AhR mRNA in Swan 71
Relative mRNA expression
p = 0.0019
p = 0.0028
p = 0.0008
Vector
OE-AhR
NC
si-AhR#1
si-AhR#2
B
AhR mRNA in HTR-8/SVneo
Relative mRNA expression
p = 0.0039
p = 0.019
p = 0.029
Vector
AhR
NC
si-AhR#1
si-AhR#2
C
Swan 71
HTR-8/SVneo
Relative mRNA expression
BRCA1
Vector
OE-AhR
NC
si-AhR#1
si-AhR#2
D
Swan 71
HTR-8/SVneo
AhR
BRCA1
H3
β-Tubulin
96 kDa
207 kDa
17 kDa
55 kDa
N C N C N C N C
Vector OE-AhR Vector OE-AhR
Protein expression level (ratio to β-Tubulin/H3%)
Cytoplasm
Nucleus
AhR BRCA1
E
HR Assay in HTR-8/SVneo
Relative HR efficiency (%)
p = 0.0069
p = 0.0112
NC
si-AhR#1
si-AhR#2
F
AhR mRNA in Swan 71
Relative mRNA expression
p = 0.0025
p = 0.0176
p = 0.0015
p = 0.0076
p = 0.0037
Vector
12 h
24 h
36 h
OE-HZ10
NC
si-HZ10#1
si-HZ10#2
G
Swan 71
AhR
β-Tubulin
H3
96 kDa
55 kDa
17 kDa
N C N C N C N C N C
Vector OE-HZ10 NC si-HZ10#1 si-HZ10#2
Protein expression level (ratio to β-Tubulin/H3%)
Cytoplasm
Nucleus
Vector
OE-HZ10
NC
si-HZ10#1
si-HZ10#2
AhR
H
Swan 71
Vector
OE-HZ10
+CHX 0 2 4 6 8 0 2 4 6 8 (h)
AhR
β-Tubulin
NC
si-HZ10
I
Swan 71
HTR-8/SVneo
AhR
β-Tubulin
96 kDa
55 kDa
Control
MG132
J
CUL4B in Swan 71
Relative mRNA expression
p = 0.0039
p = 0.0027
p = 0.0033
Vector
OE-CUL4B
NC
si-CUL4B#1
si-CUL4B#2
K
Swan 71
CUL4B
β-Tubulin
103 kDa
55 kDa
Vector
OE-CUL4B
NC
si-CUL4B#1
si-CUL4B#2
L
ChIP in HTR-8/SVneo
IgG
AhR
BRCA1 promoter binding relative fold change
p < 0.0001
p < 0.0001
p = 0.0055
Vector
OE-HZ10
M
Swan 71
HTR-8/SVneo
OE-HZ10
Si-AhR
N C
BRCA1
H3
β-Tubulin
N
Swan 71
HTR-8/SVneo
BRCA1
Protein expression level (ratio to β-Tubulin/H3%)
Cytoplasm
Nucleus
OE-HZ10
si-AhR
O
Swan 71
DAPI
BRCA1
Merge
Vector
OE-HZ10
OE-HZ10 + si-AhR
P
HTR-8/SVneo
DAPI
BRCA1
Merge
Vector
OE-HZ10
OE-HZ10 + si-AhR

Figure. S2. Lnc-HZ10 up-regulated AhR protein stability and suppressed BRCA1 transcription.

(A-B) RT-qPCR analysis of AhR mRNA levels in Swan 71 **(A)** or HTR-8/SVneo **(B)** cells with overexpression or knockdown of AhR. **(C)** RT-qPCR analysis of BRCA1 mRNA levels in Swan 71 and HTR-8/SVneo cells with overexpression or knockdown of AhR. **(D)** Nuclear/cytoplasmic fractionation assay analysis and the relative quantification of AhR and BRCA1 protein levels in the nucleus and cytoplasm of Swan 71 cells with overexpression of AhR, with β-Tubulin as cytoplasm marker and H3 as nucleus marker. **(E)** Flow cytometry analysis of relative HR efficiency in HTR-8/SVneo cells with knockdown of AhR. **(F)** RT-qPCR analysis of AhR mRNA levels in Swan 71 with overexpression of lnc-HZ10 for 12, 24, or 36 hours or knockdown of lnc-HZ10. **(G)** Nuclear/cytoplasmic fractionation assay analysis and the relative quantification of AhR protein levels in the nucleus and cytoplasm of Swan 71 cells with overexpression or knockdown of lnc-HZ10, with β-Tubulin as cytoplasm marker and H3 as nucleus marker. **(H)** Western blot analysis of AhR protein stability in lnc-HZ10-overexpressed or -silenced Swan 71 cells after CHX treatment for 0, 2, 4, 6, or 8 h, with β-Tubulin as loading control. **(I)** Western blot analysis of AhR protein levels in Swan 71 or HTR-8/SVneo cells with MG132 treatment, with β-Tubulin as loading control. **(J** and **K)** The mRNA (H) and protein (L) levels of CUL4B in Swan 71 cells with overexpression or knockdown of CUL4B. β-Tubulin protein band was used as loading control. **(L)** PCR analysis of the levels of BRCA1 promoter region enriched by AhR protein in lnc-HZ10-overexpressed HTR-8/SVneo

cells in IP assays, with IgG as negative control. **(M-N)** Nuclear/cytoplasmic fractionation assay analysis (M) and the relative quantification (N) of BRCA1 protein levels in the nucleus and cytoplasm of Swan 71 or HTR-8/SVneo cells with overexpression of lnc-HZ10 and/or knockdown of AhR, with β-Tubulin as cytoplasm marker and H3 as nucleus marker. **(O-P)** Immunofluorescence image analysis and the relative quantification of BRCA1 protein foci in Swan 71 (K) or HTR-8/SVneo (L) cells with overexpression of lnc-HZ10 and/or knockdown of AhR. Scale bar = 50 μm.

Representative data in (D, G-I, K, M, O-P) represent three independent experiments. Data in (A-C, D right, E, F, G right, J, L, N) show mean ± SD of three independent experiments. Kruskal-Wallis test for (A, B left, J left); Kruskal-Wallis test followed by Wilcoxon pairwise comparisons for (A-B right, C, D right, E, F, G right, H right, J right, L, N). *, $p < 0.05$; **, $p < 0.01$; $p < 0.05$ was considered as significant.

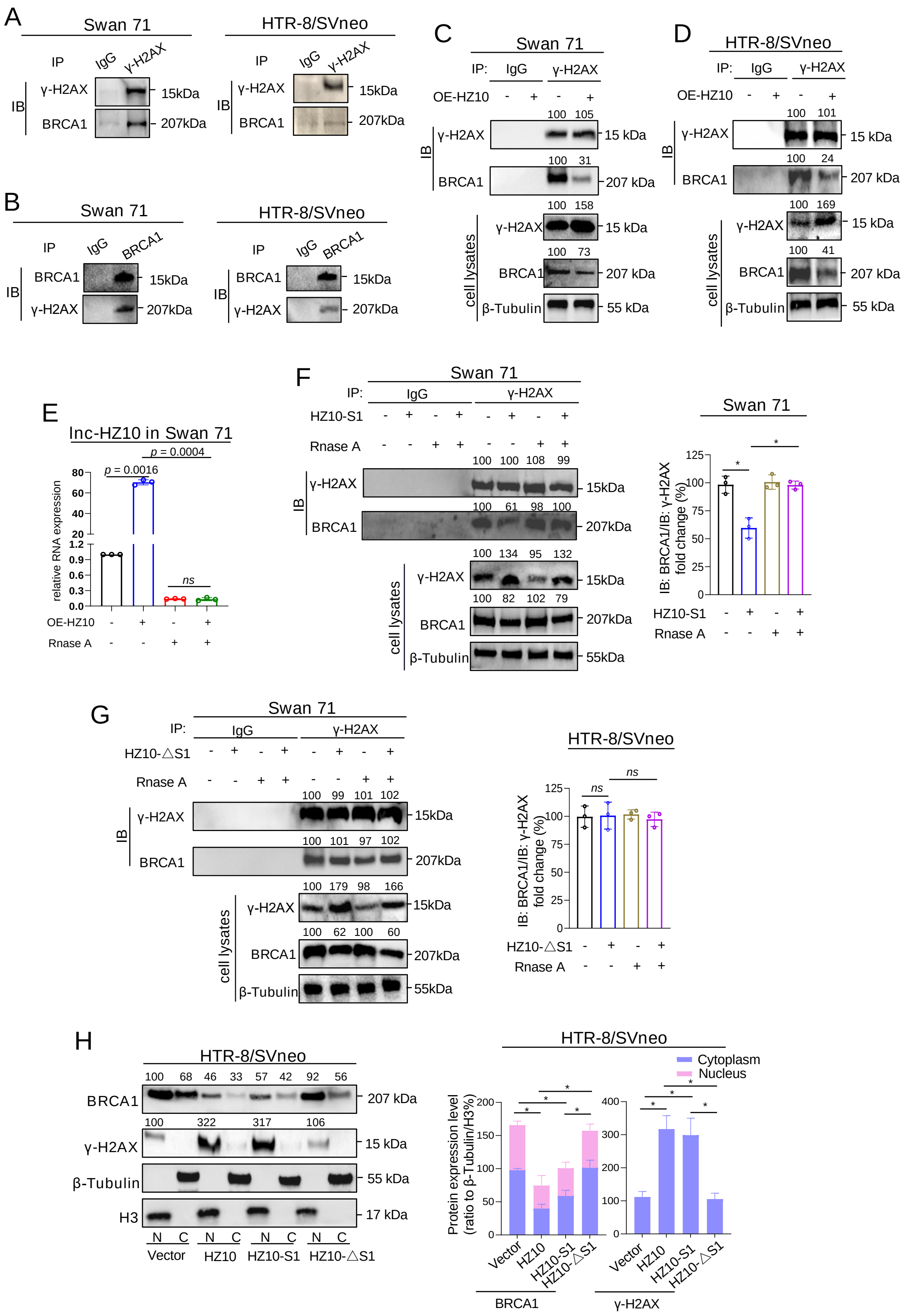

Figure. S3. Lnc-HZ10 impaired protein interactions between γ-H2AX and BRCA1.

(A-B) Western blot analysis of the levels of BRCA1 protein that was pulled down by

γ-H2AX and the levels of γ-H2AX protein that was pulled down by BRCA1 in Swan 71 or HTR-8/SVneo cells , with IgG as negative control. **(C-D)** Western blot analysis of the levels of BRCA1 protein that was immunoprecipitated by γ-H2AX in Swan 71 (C) or HTR-8/SVneo (D) cells in IP assays using a limited and identical amount of γ-H2AX antibody, with IgG as negative control. **(E)** RT-qPCR analysis of lnc-HZ10 levels in lnc-HZ10-overexpressed Swan 71 cell lysates treated with Rnase A. **(F-G)** Western blot analysis and the relative quantification of the levels of BRCA1 protein that was pulled down by γ-H2AX in lnc-HZ10-S1- (F) or lnc-HZ10-ΔS1- (G) overexpressed Swan 71 cell lysates treated with Rnase A in IP assays using a limited and identical amount of γ-H2AX antibody, with IgG as negative control. **(H)** Nuclear/cytoplasmic fractionation assay analysis and the relative quantification of BRCA1 and γ-H2AX protein levels in lnc-HZ10-, lnc-HZ10-S1-, or lnc-HZ10-ΔS1-overexpressed HTR-8/SVneo cells, with β-Tubulin as cytoplasm marker and H3 as nucleus marker.

Representative data in (A-D, F-H left) represent three independent experiments. Data in (E) show mean ± SD of three independent experiments. Kruskal-Wallis test followed by Wilcoxon pairwise comparisons for (E, F-H right). *, $p < 0.05$; **, $p < 0.01$; $p < 0.05$ was considered as significant.

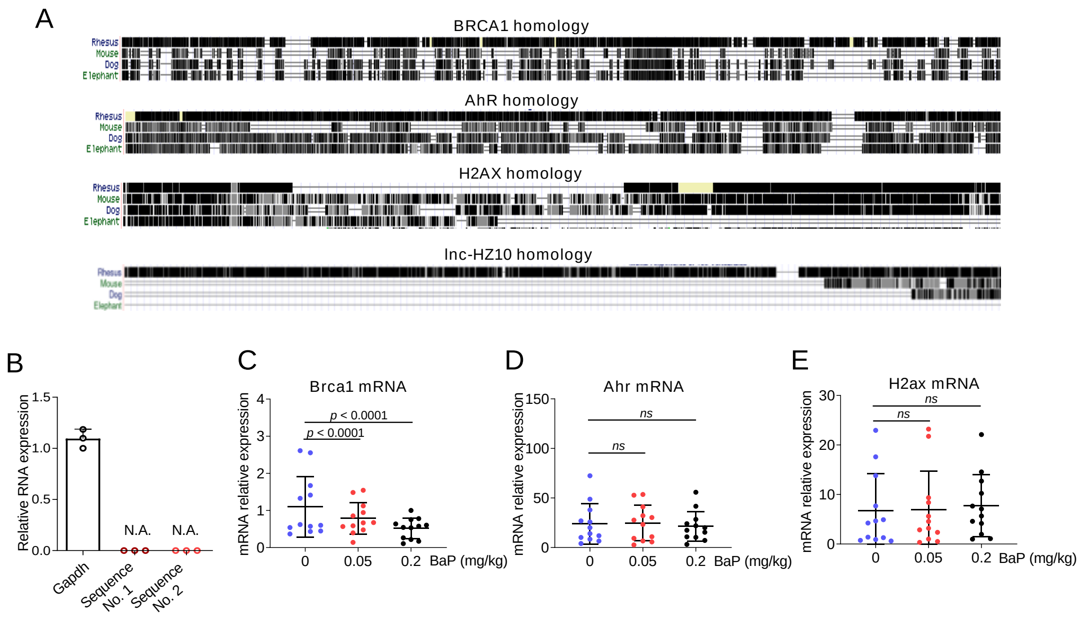

Figure. S4. Sequence conservation analysis and murine Brca1, Ahr, and H2ax mRNA levels in BaP-exposed mouse placental tissues.

(A) The sequence conservation analysis of BRCA1, AhR, H2AX, and lnc-HZ10 in human with various species, including rhesus, mouse, dog, and elephant. **(B)** RT-qPCR analysis of the levels of two fragments with similar sequence to human lnc-HZ10 in mouse placental tissues. **(C-E)** RT-qPCR analysis of the mRNA levels of murine Brca1 **(C)**, Ahr **(D),** and H2ax **(E)** in placental tissues of BaP-exposed mice (each n = 12).

Data in (B-E) show mean ± SD of 12 independent samples. Kruskal-Wallis test for (B); one-way ANOVA tests followed by Tukey's multiple comparisons test for (C-E).

Reference

[1] V. Tripathi, J. D. Ellis, Z. Shen, D. Y. Song, Q. Pan, A. T. Watt, S. M. Freier, C. F. Bennett, A. Sharma, P. A. Bubulya, B. J. Blencowe, S. G. Prasanth, and K. V. Prasanth, *Mol. Cell.* **2010**, 39, 925.
